# Racial and Ethnic Disparities in Access to Minimally Invasive Mitral Valve Surgery

**DOI:** 10.1001/jamanetworkopen.2022.47968

**Published:** 2022-12-21

**Authors:** Laurent G. Glance, Karen E. Joynt Maddox, Michael Mazzefi, Peter W. Knight, Michael P. Eaton, Changyong Feng, Miklos D. Kertai, James Albernathy, Isaac Y. Wu, Julie A. Wyrobek, Marisa Cevasco, Nimesh Desai, Andrew W. Dick

**Affiliations:** 1 Department of Anesthesiology and Perioperative Medicine, University of Rochester School of Medicine, Rochester, New York; 2 Department of Public Health Sciences, University of Rochester School of Medicine, Rochester, New York; 3 RAND Health, RAND, Boston, Massachusetts; 4 Department of Medicine, Washington University in St. Louis, St. Louis, Missouri; 5 Center for Health Economics and Policy at the Institute for Public Health, Washington University in St Louis, St Louis, Missouri; 6 Department of Anesthesiology, University of Virginia School of Medicine, Charlottesville; 7 Department of Surgery, University of Rochester School of Medicine, Rochester, New York; 8 Department of Biostatistics and Computational Biology, University of Rochester School of Medicine, Rochester, New York; 9 Department of Anesthesiology, Vanderbilt University Medical Center, Nashville, Tennessee; 10 Department of Anesthesiology and Critical Care Medicine, Johns Hopkins, Baltimore, Maryland; 11 Department of Surgery, University of Pennsylvania School of Medicine, Philadelphia

## Abstract

**Importance:**

Whether people from racial and ethnic minority groups experience disparities in access to minimally invasive mitral valve surgery (MIMVS) is not known.

**Objective:**

To investigate racial and ethnic disparities in the utilization of MIMVS.

**Design, Setting, and Participants:**

This cross-sectional study used data from the Society of Thoracic Surgeons Database for patients who underwent mitral valve surgery between 2014 and 2019. Statistical analysis was performed from January 24 to August 11, 2022.

**Exposures:**

Patients were categorized as non-Hispanic White, non-Hispanic Black, and Hispanic individuals.

**Main Outcomes and Measures:**

The association between MIMVS (vs full sternotomy) and race and ethnicity were evaluated using logistic regression.

**Results:**

Among the 103 753 patients undergoing mitral valve surgery (mean [SD] age, 62 [13] years; 47 886 female individuals [46.2%]), 10 404 (10.0%) were non-Hispanic Black individuals, 89 013 (85.8%) were non-Hispanic White individuals, and 4336 (4.2%) were Hispanic individuals. Non-Hispanic Black individuals were more likely to have Medicaid insurance (odds ratio [OR], 2.21; 95% CI, 1.64-2.98; *P* < .001) and to receive care from a low-volume surgeon (OR, 4.45; 95% CI, 4.01-4.93; *P* < .001) compared with non-Hispanic White individuals. Non-Hispanic Black individuals were less likely to undergo MIMVS (OR, 0.65; 95% CI, 0.58-0.73; *P* < .001), whereas Hispanic individuals were not less likely to undergo MIMVS compared with non-Hispanic White individuals (OR, 1.08; 95% CI, 0.67-1.75; *P* = .74). Patients with commercial insurance had 2.35-fold higher odds of undergoing MIMVS (OR, 2.35; 95% CI, 2.06-2.68; *P* < .001) than those with Medicaid insurance. Patients operated by very-high volume surgeons (300 or more cases) had 20.7-fold higher odds (OR, 20.70; 95% CI, 12.7-33.9; *P* < .001) of undergoing MIMVS compared with patients treated by low-volume surgeons (less than 20 cases). After adjusting for patient risk, non-Hispanic Black individuals were still less likely to undergo MIMVS (adjusted OR [aOR], 0.88; 95% CI, 0.78-0.99; *P* = .04) and were more likely to die or experience a major complication (aOR, 1.25; 95% CI, 1.16-1.35; *P* < .001) compared with non-Hispanic White individuals.

**Conclusions and Relevance:**

In this cross-sectional study, non-Hispanic Black patients were less likely to undergo MIMVS and more likely to die or experience a major complication than non-Hispanic White patients. These findings suggest that efforts to reduce inequity in cardiovascular medicine may need to include increasing access to private insurance and high-volume surgeons.

## Key Points

**Question:**

Do non-Hispanic Black and Hispanic individuals experience disparities in access to minimally invasive mitral valve surgery?

**Findings:**

In this cross-sectional study of 103 753 patients, non-Hispanic Black individuals were less likely to undergo minimally invasive mitral valve surgery, whereas Hispanic individuals had similar rates of minimally invasive surgery compared with non-Hispanic White individuals. Non-Hispanic Black individuals were more likely than non-Hispanic White individuals to have Medicaid insurance and to be treated by low-volume surgeons, both factors being associated with lower rates of minimally invasive surgery.

**Meaning:**

These findings suggest that efforts to improve equity in cardiovascular medicine may need to include expanding access to commercial insurance and high-volume surgeons.

## Teaser

This cross-sectional study examines racial and ethnic disparities in the utilization of minimally invasive mitral valve surgery.

## Introduction

Race-based disparity may be the most common cause of death among Black men and women under age 65 years of age.^1,2^ Cardiovascular disease accounts for more than one-third of the mortality difference between Black and White individuals in the US^3^ and remains the number 1 cause of death in the US.^4^ Cardiovascular procedures are the most common inpatient surgical procedures in adults aged 45 to 64 years and 75 years and older in the US.^5^ Despite the effectiveness of cardiovascular interventions, adults from racial and ethnic minority groups and those with low incomes are less likely to receive these interventions and are more likely to have poorer outcomes after undergoing these therapies. For example, Black patients with coronary heart disease are less likely than White patients to undergo cardiac catheterization, percutaneous coronary intervention, or surgical revascularization.^6,7,8,9,10^ Black patients with severe aortic stenosis are less likely than White patients to undergo aortic valve replacement.^11^ Adults from racial and ethnic minority groups and with low incomes who do undergo surgical revascularization or heart valve surgery are more likely to receive care from lower quality, lower volume hospitals and surgeons, and have higher periprocedural mortality.^8,12,13,14,15,16,17,18,19,20^

One potential mechanism underlying these differences in outcomes could be related to the surgical approach. For many noncardiac surgical procedures, minimally invasive approaches are associated with less pain, decreased morbidity, and a faster recovery, and are increasingly considered the standard of surgical care.^21,22,23,24,25^ For cardiac surgery, in particular, the use of minimally invasive approaches to mitral valve disease is increasing.^26,27^ Minimally invasive mitral valve surgery may be associated with a lower risk of surgical site complications and mortality, a similar need for reoperation, faster recovery, better functional outcomes, and higher patient satisfaction rates than conventional sternotomy.^28,29,30,31,32^

However, although racial disparities in the use of minimally invasive surgery have been reported for noncardiac surgery,^33^ disparities in utilization of and outcomes after minimally invasive approaches for mitral valve surgery have not been examined. Our primary goal was to examine racial and ethnic disparities in the utilization and outcomes of minimally invasive mitral valve surgery. We also sought to determine whether people from racial and ethnic minority groups were more likely to undergo mitral valve surgery with low-volume surgeons because, for many surgical procedures, there is a well-described association between higher case volumes and better outcomes.^34,35^

## Methods

### Study Approval

The University of Rochester research study review board reviewed this study and determined that it met federal and university criteria for exemption because it consisted of secondary research on existing data. Therefore, informed consent was not required. This study was approved by the Society of Thoracic Surgeons (STS) Research Center and followed the Strengthening the Reporting of Observational Studies in Epidemiology (STROBE) reporting guideline.^36^

### Data Source

This retrospective cross-sectional study was conducted using preexisting patient-level data from the STS National Adult Cardiac Surgery Database (ACSD). The data for this research were provided by the STS National Database Participant User File Research Program. Data analysis was performed at the University of Rochester. The ACSD contains more than 7.4 million patient records from 2903 surgeons and has been used for public and nonpublic performance assessment, quality improvement, and comparative effectiveness research.^37^ These data have been used in many prior studies.^18,19,38^ The database includes information on patient demographics (age, sex, race, ethnicity, height, and weight), payer status (Medicaid, self-paid, Medicare, health maintenance organization [HMO], commercial), urgency (elective, urgent, emergent, salvage), severity-of-disease (ejection fraction, heart failure, prior myocardial infarction, valvular heart disease, mechanism of mitral regurgitation), comorbidities (stroke, kidney function, lung disease, atrial fibrillation), surgical approach (sternotomy, less invasive), encrypted surgeon and hospital identifiers, and outcomes (mortality, complications). Race and ethnicity are self-reported by the patient or by the family.

### Study Population

We identified 121 709 patients undergoing planned isolated mitral valve repair or replacement (MVRR) between 2014 and 2019. We excluded patients on extracorporeal membrane oxygenation or with mechanical circulatory support before surgery (n = 277), percutaneous approaches (n = 1216), operative approach missing (n = 235), other operative approach (n = 239), race not Black or White (n = 7942), race missing (n = 2701), Hispanic ethnicity missing (n = 4101), and payer missing (n = 962). The full list of exclusions is shown in the flow diagram (eFigure 1 in Supplement 1). The final data set consisted of 103 753 cases by 2690 surgeons at 1085 hospitals.

### Statistical Analysis 

#### Utilization of Minimally Invasive Surgery

We used multivariable logistic regression to estimate the association between race and ethnicity and the use of a minimally invasive approach for isolated mitral valve repair or replacement (MVRR). The dependent variable was the surgical approach: minimally invasive vs full sternotomy. We defined the minimally invasive approach when the operative approach was coded as either a thoracotomy, partial sternotomy, parasternal incision, or sub-xyphoid approach. In this intention-to-treat analysis, procedures converted from a minimally invasive to a standard approach were considered minimally invasive. The exposure was race and ethnicity categorized as follows: (1) Hispanic, (2) non-Hispanic Black, and (3) non-Hispanic White. We treated the findings from the unconditional analyses (in which we did not adjust for patient risk factors) as the main findings. Non-Hispanic Black and Hispanic patients may present for surgery with more advanced disease and greater comorbidity burden because of the impact of social determinants of health and structural racism. Hence, adjusting for patient disease may underestimate the magnitude of disparities.^39^

We estimated a nonparsimonious model in which race and ethnicity was the exposure variable, and in which we controlled for age; sex; body mass index (BMI, calculated as weight in kilograms divided by height in meters squared) (underweight [BMI: <18.5], normal weight [BMI: 18.5-24.9], overweight [BMI: 25.0-29.9], obese [BMI: 30.0-39.9], morbid obesity [BMI: ≥40.0]); surgical urgency (elective, urgent, emergent), prior myocardial infarction (less than 24 hours, 1 to 7 days, 8 to 21 days, more than 21 days), aortic insufficiency (trivial, mild, moderate, severe), chronic kidney disease (mildly decreased [stage 2: glomerular filtration rate [GFR], 60-89 mL/min], mildly to moderately decreased [stage 3A: GFR, 45-59], moderately to severely decreased [stage 3B: GFR, 30-44 mL/min], severely decreased [stage 4: GFR, 15-29 mL/min], kidney failure [stage 5: GFR, <15 mL/min]); lung disease (mild, moderate, severe), pneumonia (remote [more than 1 month prior to procedure], recent [within 1 month of procedure]); stroke (remote [more than 1 month prior to procedure], recent [within 1 month of procedure]); liver disease; atrial fibrillation; mechanism for mitral insufficiency (myxomatous, rheumatic, functional, infectious endocarditis); prior cardiac surgery (prior coronary artery bypass graft, prior valve surgery, prior coronary artery bypass graft and valve surgery); preoperative intraaortic balloon pump, and year of surgery. We then estimated sequential models (eTable 2 in Supplement 1) to examine the extent to which controlling for payer status, the hospital proportion of non-Hispanic Black patients undergoing mitral valve surgery, and surgeon case volume would attenuate the association between race and ethnicity and the use of a minimally invasive approach: (1) model 2: baseline model plus payer status (Medicaid, self-paid, Medicare, HMO, commercial, other); (2) model 3: baseline model plus payer status plus hospital proportion of non-Hispanic Black patients (less than 5%, 5.0% to 9.9%, 10.0% to 19.9%, 20.0% to 29.9%, 30.0% or more) plus surgeon case volume. We chose to characterize high-minority hospitals using the proportion of non-Hispanic Black patients undergoing mitral valve surgery because racial and ethnic disparities in the use of minimally invasive surgery were much more pronounced for non-Hispanic Black patients than for Hispanic patients. For the purpose of our analyses, we specified patients with both Medicare and Medicaid coverage (ie, dual-eligible) as having Medicaid coverage.

#### Outcomes After Minimally Invasive Surgery

We also examined the association between the composite outcome of inpatient and 30-day mortality and major morbidity as defined by the STS (stroke, kidney failure, cardiac reoperation, deep sternal wound infection, prolonged ventilation) and race and ethnicity. We also performed additional analyses in which we interacted race and ethnicity with payer status to determine whether the association between the use of minimally invasive surgery was different by race and ethnicity within each of the payer groups (eg, commercial insurance). We then examined the interaction between race and ethnicity and (1) hospital proportion of non-Hispanic Black patients and (2) surgeon case volume. We also examined the interaction of race and ethnicity and surgeon case volume for the composite outcome.

Finally, we used logistic regression analysis to examine the association between patient race and ethnicity and (1) payer status, (2) hospitalization in a Black-serving hospital, and (3) treatment by a high-volume surgeon. These analyses were unadjusted except for payer status, where we adjusted for patient age.

We used multivariate multiple imputation (mi impute chained) with chained equations (MICE) to impute missing data. We specified multinomial logistic regression models for categorical variables and logistic regression models for binary variables.

All statistical analyses were performed using STATA/MP version 17.0 (StataCorp LLC) from January 24 to August 11, 2022. We used cluster robust variance estimators to account for the clustering of observations within hospitals. We estimated adjusted rates and outcomes using average marginal effects. The threshold for statistical significance was a 2-sided *P* < .05.

## Results

### Patient Population

The study was based on data from 103 753 surgical procedures (Table 1; eFigure 2 in Supplement 1). Among these patients, 47 886 (46.2%) were women; 4336 (4.2%) were Hispanic individuals; 10 404 (10.0%) were non-Hispanic Black individuals; 89 013 (85.8%) were non-Hispanic White individuals; and the mean (SD) age was 62.4 (13.0) years (Table 1). Non-Hispanic White individuals were more likely to have commercial insurance (32.8% [n = 29 167]) compared with non-Hispanic Black (24.2% [n = 2517]) and Hispanic individuals (23.0% [n = 997]). Non-Hispanic White individuals were less likely to have Medicaid insurance (6.3% [n = 5609]) compared with non-Hispanic Black (22.5% [n = 2340]) and Hispanic individuals (20.3% [n = 878]), and less likely to be self-paid (2.5% [n = 2199]) compared with non-Hispanic Black (5.8% [n = 602]) and Hispanic (5.5% [n = 240]) individuals. Non-Hispanic White individuals were less likely to have obesity (23.2% [n = 20 665]) and morbid obesity (3.8% [n = 3369]) compared with non-Hispanic Black (obesity: 30.6% [n = 3180]; morbid obesity: 9.5% [n = 990]) and Hispanic (obesity: 29.6% [n = 1283]; morbid obesity: 5.0% [n = 217]) individuals. Non-Hispanic White individuals were less likely to have a history of congestive heart failure (54.2% [n = 48 220]) compared with non-Hispanic Black (69.7% [n = 7257]) and Hispanic (59.7% [n = 2859]) individuals, and less likely to have kidney failure (1.6% [n = 1433]) compared with non-Hispanic Black (10.4% [n = 1086]) and Hispanic (5.3% [n = 229]) individuals.

**Table 1.** Patient Characteristics

Characteristic	Patients, No. (%)				*P* value
	All	Non-Hispanic White	Non-Hispanic Black	Hispanic	
No.	103 753	89 013 (85.8)	10 404 (10)	4336 (4.2)	
Payer					
Medicaid	8827 (8.5)	5609 (6.3)	2340 (22.5)	878 (20.3)	<.001
Self-paid	3041 (2.9)	2199 (2.5)	602 (5.8)	240 (5.5)	
Medicare	40 983 (39.5)	36 882 (41.4)	2938 (28.2)	1163 (26.8)	
HMO	13 456 (13)	11 330 (12.7)	1301 (12.5)	825 (19)	
Other	4765 (4.6)	3826 (4.3)	706 (6.8)	233 (5.4)	
Commercial	32 681 (31.5)	29 167 (32.8)	2517 (24.2)	997 (23.0)	
Age, y					
<40	6664 (6.4)	4847 (5.5)	1408 (13.5)	409 (9.4)	<.001
41-50	10 515 (10.1)	7985 (9)	1950 (18.7)	580 (13.4)	
51-60	24 450 (23.6)	20 270 (22.8)	3129 (30.1)	1051 (24.2)	
61-70	32 150 (31)	28 395 (31.9)	2614 (25.1)	1141 (26.3)	
71-80	23 920 (23.1)	21 861 (24.6)	1126 (10.8)	933 (21.5)	
≥81	6054 (5.8)	5655 (6.4)	177 (1.7)	222 (5.1)	
Sex					
Male	55 867 (53.9)	49 380 (55.5)	4408 (42.4)	2079 (48)	<.001
Female	47 886 (46.2)	39 633 (44.5)	5996 (57.6)	2257 (52.1)	
BMI					
<18.5	2534 (2.4)	2157 (2.4)	309 (3)	68 (1.6)	<.001
18.5-24.9	35 028 (33.8)	31 090 (34.9)	2764 (26.6)	1174 (27.1)	
25-25.9	36 487 (35.2)	31 732 (35.7)	3161 (30.4)	1594 (36.8)	
30-39.9	25 128 (24.2)	20 665 (23.2)	3180 (30.6)	1283 (29.6)	
≥40	4576 (4.4)	3369 (3.8)	990 (9.5)	217 (5.0)	
Surgical urgency					
Elective	80 396 (77.5)	70 683 (79.4)	6677 (64.2)	3036 (70)	<.001
Urgent	22 065 (21.3)	17 263 (19.4)	3555 (34.2)	1247 (28.8)	
Emergent	1198 (1.2)	984 (1.1)	166 (1.6)	48 (1.1)	
Salvage	94 (0.1)	83 (0.1)	6 (0.1)	5 (0.1)	
Transfer from other hospital					
None	92 874 (89.5)	79 949 (89.8)	9101 (87.5)	3824 (88.2)	<.001
Transfer	9039 (8.7)	7290 (8.2)	1265 (12.2)	484 (11.2)	
Missing	1840 (1.8)	1774 (2)	38 (0.4)	28 (0.7)	
Shock	381 (0.37)	306 (0.34)	55 (0.53)	20 (0.46)	.007
Ejection fraction					
≥60%	54 974 (53)	48 485 (54.5)	4439 (42.7)	2050 (47.3)	<.001
50%-59.9%	31 402 (30.3)	27 126 (30.5)	2923 (28.1)	1353 (31.2)	
40%-49.9%	8758 (8.4)	7014 (7.9)	1307 (12.6)	437 (10.1)	
30%-39.9%	4516 (4.4)	3223 (3.6)	1009 (9.7)	284 (6.6)	
20%-29.9%	1824 (1.8)	1263 (1.4)	453 (4.4)	108 (2.5)	
<20%	250 (0.2)	169 (0.2)	66 (0.6)	15 (0.4)	
Missing	2029 (2)	1733 (2)	207 (2)	89 (2.1)	
Prior myocardial infarction					
None	93 885 (90.5)	80 898 (90.9)	9130 (87.8)	3857 (89)	<.001
>21 d	8234 (7.9)	6831 (7.7)	1009 (9.7)	394 (9.1)	
8-21 d	708 (0.7)	530 (0.6)	137 (1.3)	41 (1)	
1-7 d	763 (0.7)	614 (0.7)	112 (1.1)	37 (0.9)	
<24 h	163 (0.2)	140 (0.2)	16 (0.2)	7 (0.2)	
CHF					
None	45 687 (44)	40 793 (45.8)	3147 (30.3)	1747 (40.3)	<.001
>2 wk	37 953 (36.6)	31 440 (35.3)	4820 (46.3)	1693 (39.1)	
≤2 wk	20 113 (19.4)	16 780 (18.9)	2437 (23.4)	896 (20.7)	
Aortic insufficiency					
None	60 410 (58.2)	51 515 (57.9)	6418 (61.7)	2477 (57.1)	<.001
Trivial	19 753 (19)	17 332 (19.5)	1672 (16.1)	749 (17.3)	
Mild	14 974 (14.4)	12 957 (14.6)	1297 (12.5)	720 (16.6)	
Moderate	3168 (3.1)	2675 (3)	339 (3.3)	154 (3.6)	
Severe	161 (0.2)	120 (0.1)	30 (0.3)	11 (0.3)	
Missing	5287 (5.1)	4414 (5)	648 (6.2)	225 (5.2)	
Glomerular filtration rate					
Normal, (≥90 mL/min)	20 462 (19.7)	16 340 (18.4)	3101 (29.8)	1021 (23.6)	<.001
Mildly decreased (60-89 mL/min)	54 459 (52.5)	48 436 (54.4)	4061 (39)	1962 (45.3)	
Mildly to moderately decreased (45-59 mL/min)	16 784 (16.2)	14 868 (16.7)	1259 (12.1)	657 (15.2)	
Moderately to severely decreased (30-44 mL/min)	7316 (7.1)	6322 (7.1)	640 (6.2)	354 (8.2)	
Severely decreased (15-29 mL/min)	1770 (1.7)	1433 (1.6)	241 (2.3)	96 (2.2)	
Kidney failure (<15 mL/min)	2748 (2.7)	1433 (1.6)	1086 (10.4)	229 (5.3)	
Missing	214 (0.2)	181 (0.2)	16 (0.2)	17 (0.4)	
Lung disease					
None	77 874 (75.1)	67 766 (76.1)	6807 (65.4)	3301 (76.1)	<.001
Mild	10 301 (9.9)	8586 (9.7)	1340 (12.9)	375 (8.7)	
Moderate	4644 (4.5)	3786 (4.3)	660 (6.3)	198 (4.6)	
Severe	4293 (4.1)	3472 (3.9)	649 (6.2)	172 (4)	
Severity unknown	5028 (4.9)	4114 (4.6)	720 (6.9)	194 (4.5)	
Missing	1613 (1.6)	1289 (1.5)	228 (2.2)	96 (2.2)	
Home oxygen					
None	99 108 (95.5)	85 011 (95.5)	9953 (95.7)	4144 (95.6)	<.001
Partial	1632 (1.6)	1408 (1.6)	158 (1.5)	66 (1.5)	
Oxygen-dependent	1565 (1.5)	1304 (1.5)	204 (2)	57 (1.3)	
Missing	1448 (1.4)	1290 (1.5)	89 (0.9)	69 (1.6)	
Pneumonia					
None	88 106 (84.9)	75 736 (85.1)	8702 (83.6)	3668 (84.6)	<.001
Remote	8144 (7.9)	7002 (7.9)	843 (8.1)	299 (6.9)	
Recent	4820 (4.7)	3847 (4.3)	693 (6.7)	280 (6.5)	
Missing	2683 (2.6)	2428 (2.7)	166 (1.6)	89 (2.1)	
Stroke					
None	93 767 (90.4)	81 094 (91.1)	8834 (84.9)	3839 (88.5)	<.001
>30 d	7207 (7)	5715 (6.4)	1128 (10.8)	364 (8.4)	
≤30 d	2199 (2.1)	1728 (1.9)	357 (3.4)	114 (2.6)	
Missing	580 (0.6)	476 (0.5)	85 (0.8)	19 (0.4)	
Liver disease					
No liver disease	98 560 (95)	84 852 (95.3)	9649 (92.7)	4059 (93.6)	<.001
Present	4545 (4.4)	3610 (4.1)	699 (6.7)	236 (5.4)	
Missing	648 (0.6)	551 (0.6)	56 (0.5)	41 (1)	
Atrial fibrillation					
None	72 627 (70)	61 599 (69.2)	8013 (77)	3015 (69.5)	<.001
Present	30 913 (29.8)	27 240 (30.6)	2357 (22.7)	1316 (30.4)	
Missing	213 (0.2)	174 (0.2)	34 (0.3)	5 (0.1)	
Mediastinal radiation					
None	101 099 (97.4)	86 625 (97.3)	10 225 (98.3)	4249 (98)	<.001
Yes	1710 (1.7)	1541 (1.7)	124 (1.2)	45 (1)	
Missing	944 (0.9)	847 (1)	55 (0.5)	42 (1)	
Peripheral vascular disease					
None	97 774 (94.2)	84 088 (94.5)	9641 (92.7)	4045 (93.3)	<.001
Present	5734 (5.5)	4735 (5.3)	731 (7)	268 (6.2)	
Missing	245 (0.2)	190 (0.2)	32 (0.3)	23 (0.5)	
Mechanism for MR					
Myxomatous degenerative	46 720 (45)	42 284 (47.5)	2962 (28.5)	1474 (34)	<.001
Rheumatic	8467 (8.2)	6284 (7.1)	1462 (14.1)	721 (16.6)	
Functional	2852 (2.8)	2101 (2.4)	610 (5.9)	141 (3.3)	
Infectious endocarditis	12 248 (11.8)	9909 (11.1)	1731 (16.6)	608 (14)	
Other	16 624 (16)	13 891 (15.6)	1938 (18.6)	795 (18.3)	
Missing	16 842 (16.2)	14 544 (16.3)	1701 (16.4)	597 (13.8)	
Prior cardiac surgery					
None	89 462 (86.2)	77 060 (86.6)	8797 (84.6)	3605 (83.1)	<.001
Prior CABG	2486 (2.4)	2210 (2.5)	163 (1.6)	113 (2.6)	
Prior valve surgery	9749 (9.4)	7936 (8.9)	1282 (12.3)	531 (12.3)	
Prior CABG and valve surgery	2056 (2)	1807 (2)	162 (1.6)	87 (2)	
Prior PCI					
None	95 600 (92.1)	82 009 (92.1)	9627 (92.5)	3964 (91.4)	.004
PCI, not acute	7620 (7.3)	6569 (7.4)	715 (6.9)	336 (7.8)	
PCI, acute	533 (0.5)	435 (0.5)	62 (0.6)	36 (0.8)	
IABP					
None	102 149 (98.5)	87 689 (98.5)	10 184 (97.9)	4276 (98.6)	<.001
Present	1604 (1.6)	1324 (1.5)	220 (2.1)	60 (1.4)	
Hospital proportion of non-Hispanic Black patients, %					
<5	46 116 (44.5)	43 257 (48.6)	1158 (11.1)	1701 (39.2)	<.001
5-9.9	25 686 (24.8)	22 129 (24.9)	2146 (20.6)	1411 (32.5)	
10-19.9	19 940 (19.2)	15 996 (18)	3150 (30.3)	794 (18.3)	
29-29.9	7393 (7.1)	5171 (5.8)	1935 (18.6)	287 (6.6)	
≥30	4618 (4.5)	2460 (2.8)	2015 (19.4)	143 (3.3)	
Surgeon case volume					
<20	8410 (8.1)	6688 (7.5)	1230 (11.8)	492 (11.4)	<.001
20-49	18 219 (17.6)	15 220 (17.1)	2095 (20.1)	904 (20.9)	
50-99	23 363 (22.5)	19 886 (22.3)	2530 (24.3)	947 (21.8)	
100-199	23 569 (22.7)	20 524 (23.1)	2204 (21.2)	841 (19.4)	
200-299	12 144 (11.7)	10 887 (12.2)	1030 (9.9)	227 (5.2)	
≥300	18 048 (17.4)	15 808 (17.8)	1315 (12.6)	925 (21.3)	
Outcomes					
Minimally invasive surgery	29 596 (28.5)	26 045 (29.3)	2208 (21.2)	1343 (31)	<.001
30-d or in-hospital mortality or morbidity	14 621 (14.1)	11 844 (13.3)	2074 (19.9)	703 (16.2)	<.001

Abbreviations: BMI, body mass index (calculated as weight in kilograms divided by height in meters squared); CABG, coronary artery bypass graft; CHF, congestive heart failure; HMO, health maintenance organization; IABP, intraaortic balloon pump; MR, mitral regurgitation; PCI, percutaneous coronary intervention.

Non-Hispanic Black patients were more likely to be seen at high-minority hospitals and to receive care from low-volume surgeons (eTable 1 in Supplement 1). Non-Hispanic Black individuals had 31-fold higher odds (OR, 30.6; 95% CI, 25.49-36.72; *P* < .001) of being treated in a hospital with a very high proportion of non-Hispanic Black individuals (30% or higher) compared with hospitals with a low proportion of non-Hispanic Black individuals (less than 5%) (eFigure 2 in Supplement 1). Non-Hispanic Black individuals had 2-fold higher odds of being treated by a low-volume surgeon (OR, 2.21; 95% CI, 1.64-2.98; *P* < .001) than a very-high volume surgeon compared with non-Hispanic White individuals (eFigure 2 in Supplement 1).

### Utilization of Minimally Invasive Surgery

**Figure 1.**
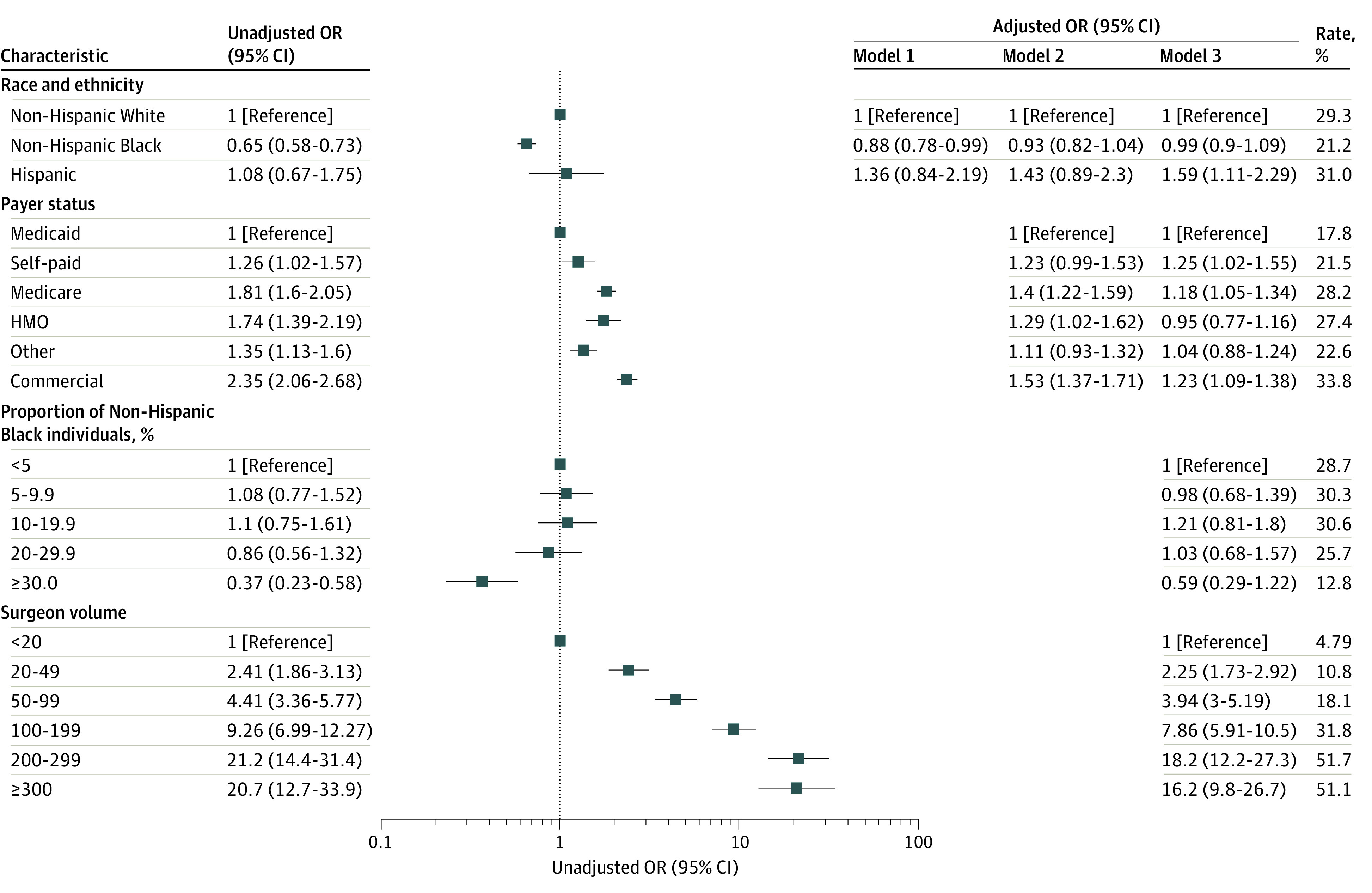
Disparities in the Utilization of Minimally Invasive Approach for Mitral Valve Surgery Odds ratio (ORs) were adjusted in model 1 for patient risk (patient demographics, surgical urgency, prior myocardial infarction, aortic insufficiency, chronic kidney disease, lung disease, pneumonia, stroke, liver disease, atrial fibrillation, infectious endocarditis, prior cardiac surgery, preoperative intraaortic balloon pump, and year of surgery); in model 2 for patient risk and payer status; and in model 3 for patient risk, payer status, hospital proportion of non-Hispanic Black patients, and surgeon case volume. The rates are based on average marginal estimates using bivariate logistic regression. HMO indicates health maintenance organization.

Non-Hispanic Black individuals were less likely to undergo minimally invasive surgery (OR, 0.65; 95% CI, 0.58-0.73; *P* < .001) compared with non-Hispanic White individuals (Figure 1). Hispanic individuals were not less likely to undergo minimally invasive surgery (OR, 1.08; 95% CI, 0.67-1.75; *P* = .74) compared with non-Hispanic White individuals.

Several other patient, hospital, and surgeon characteristics were associated with the use of minimally invasive surgery. Patients with commercial insurance had 2.35-fold higher odds of undergoing minimally invasive surgery (OR, 2.35; 95% CI, 2.06-2.68; *P* < .001) than those with Medicaid insurance. Patients in high-minority hospitals had 63% lower odds of undergoing minimally invasive surgery (OR, 0.37; 95% CI, 0.23-0.58; *P* < .001) compared with patients in low-minority hospitals (Figure 1). Patients operated by a high-volume (200 to 299 cases) and very-high volume (300 or more cases) surgeon had 21.2-fold (95% CI; 14.4-31.4; *P* < .001) and 20.7-fold (95% CI, 12.7-33.9; *P* < .001) higher odds of undergoing a minimally invasive procedure compared with patients treated by low-volume surgeons (less than 20 cases) (Figure 1).

**Figure 2.**
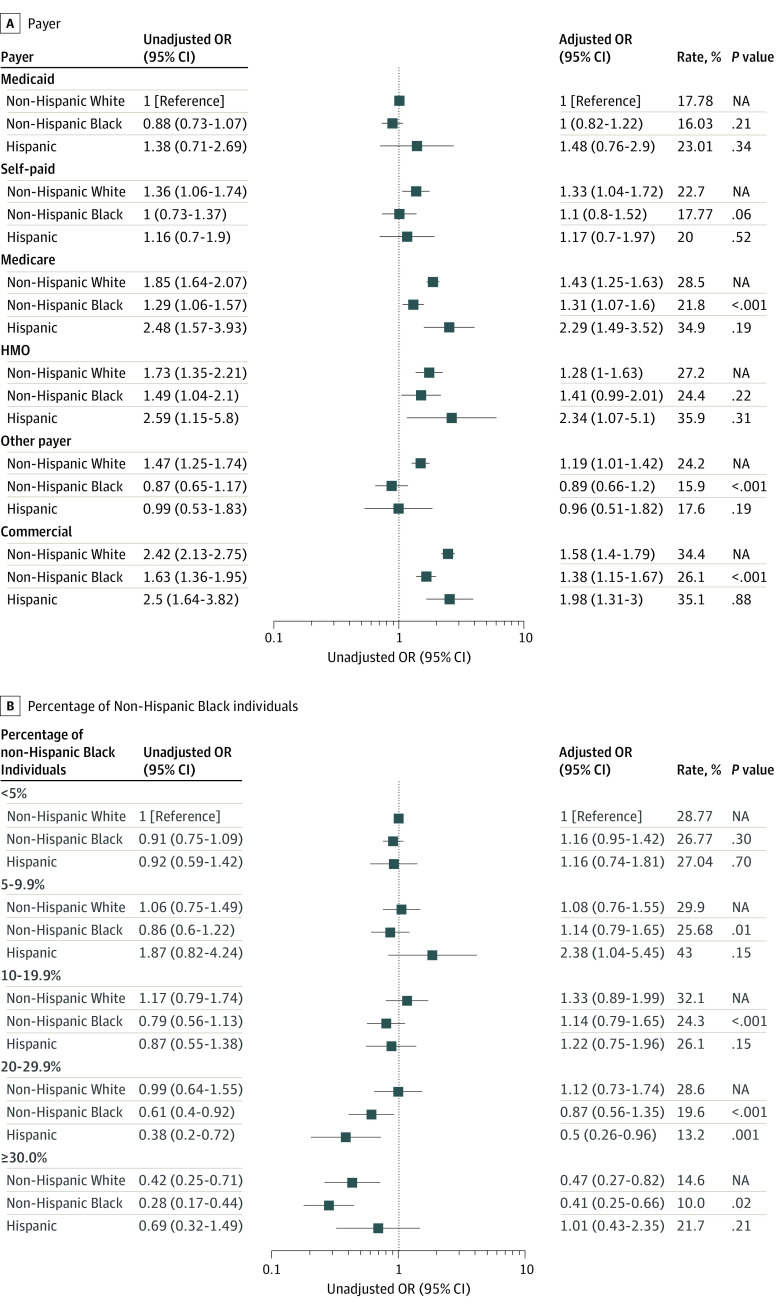
Disparities in the Utilization of Minimally Invasive Approach for Mitral Valve Surgery Within Payers and Hospitals Odds ratios (ORs) were adjusted for patient demographics, surgical urgency, prior myocardial infarction, aortic insufficiency, chronic kidney disease, lung disease, pneumonia, stroke, liver disease, atrial fibrillation, infectious endocarditis, prior cardiac surgery, preoperative intraaortic balloon pump, and year of surgery. The adjusted rates are based on average marginal estimates based on the unadjusted logistic regression model. A, Disparities within payers; *P* values are for comparisons with non-Hispanic White individuals within the same payer strata (unadjusted). B, Disparities within hospitals; *P* values are for comparisons with non-Hispanic White individuals within the same hospital strata (unadjusted). HMO indicates health maintenance organization. NA indicates not applicable.

**Figure 3.**
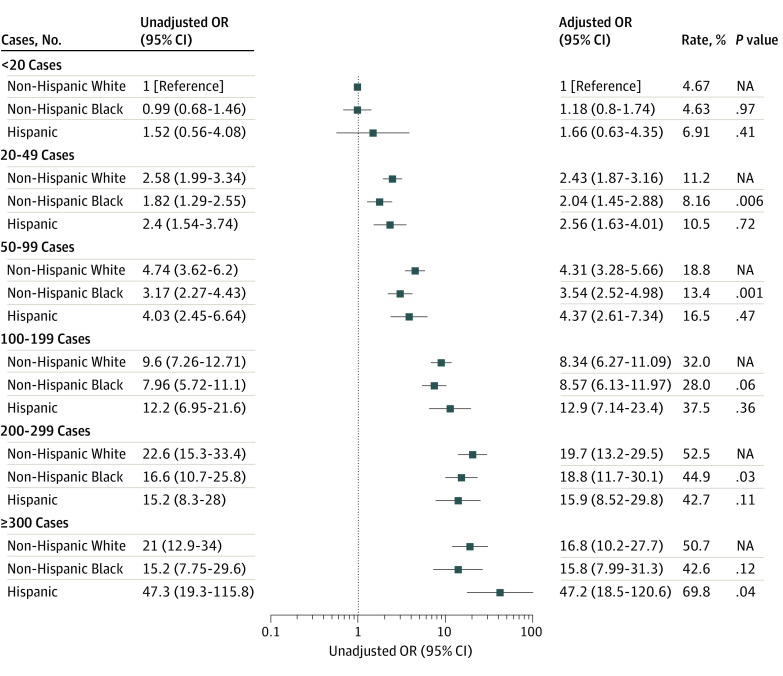
Disparities in the Utilization of Minimally Invasive Approach for Mitral Valve Surgery Within Surgeons Odds ratios (ORs) were adjusted for patient demographics, surgical urgency, prior myocardial infarction, aortic insufficiency, chronic kidney disease, lung disease, pneumonia, stroke, liver disease, atrial fibrillation, infectious endocarditis, prior cardiac surgery, preoperative intraaortic balloon pump, and year of surgery. The adjusted rates are based on average marginal estimates based on the unadjusted logistic regression model. *P* values are for comparisons with non-Hispanic White individuals within the same surgeon strata (unadjusted). NA indicates not applicable.

Even in patients with commercial insurance, non-Hispanic Black individuals were still less likely to undergo a minimally invasive approach compared with non-Hispanic White individuals (26.1% [95% CI, 22.2%-29.9%] vs 34.4% [95% CI, 30.9%-37.8%]; *P* < .001) (Figure 2A). Non-Hispanic Black individuals were less likely to undergo minimally invasive surgery in high-minority hospitals than non-Hispanic White individuals (10.0% [95% CI, 6.10%-14.0%] vs 14.6% [95% CI, 8.59%-20.7%]; *P* = .02) (Figure 2B). Non-Hispanic Black individuals were also less likely to undergo minimally invasive surgery if they were treated by high-volume (44.9% [95% CI, 35.2%-54.5%] vs 52.5% [95% CI, 44.1%-60.9%]; *P* = .03) compared with non-Hispanic White individuals (Figure 3).

After adjusting for age, sex, BMI, surgical urgency, aortic insufficiency, comorbidities, prior surgery, and intraaortic balloon pump, non-Hispanic Black individuals were still less likely to undergo minimally invasive surgery (adjusted OR [aOR], 0.88; 95% CI, 0.78-0.99; *P* = .04) compared with non-Hispanic White individuals, although the effect size was smaller than in the unadjusted analyses (Figure 1). After controlling for these factors and adjusting for payer status, there was no significant association between non-Hispanic Black individuals and minimally invasive surgery (aOR, 0.93; 95% CI, 0.82-1.04; *P* = .21) (Figure 1). Further adjusting for the hospital proportion of non-Hispanic Black individuals and surgeon case volume resulted in further attenuation of this association: aOR, 0.99; 95% CI, 0.90-1.09; *P* = .81) (Figure 1).

### Outcomes After Minimally Invasive Surgery

Compared with White individuals, non-Hispanic Black individuals had 62% higher odds (OR, 1.62; 95% CI, 1.51-1.74; *P* < .001) of death or major morbidity and Hispanic individuals had 26% higher odds (OR, 1.26; 95% CI, 1.09-1.45; *P* < .001) (Table 2). After adjusting for patient risk factors, non-Hispanic Black individuals had 25% (aOR, 1.25; 95% CI, 1.16-1.35; *P* < .001) higher odds of death or major morbidity. Hispanic individuals did not have a statistically significant greater risk of death or major morbidity (OR, 1.08; 95% CI, 0.95-1.22; *P* = .25) compared with non-Hispanic White individuals. The odds of death or complications in non-Hispanic Black individuals were unchanged after adjusting for the use of a minimally invasive approach (aOR, 1.25; 95% CI, 1.16-1.34; *P* < .001) or after adjusting for both the use of a minimally invasive approach and payer status (aOR, 1.22; 95% CI, 1.14-1.32; *P* < .001) compared with non-Hispanic White individuals. Further adjusting for the hospital proportion of non-Hispanic Black patients (aOR, 1.14; 95% CI, 1.06-1.23; *P* < .001) was associated with a slight reduction in the odds of death or major complications. Further adjusting for surgeon case volume was not associated with a decrease in the odds of mortality or major complications (aOR, 1.14; 95% CI, 1.06-1.23; *P* < .001).

**Table 2.** Thirty-Day Mortality or Morbidity

	Unadjusted		Patient-level		Patient-level + minimally invasive surgery		Patient-level + minimally invasive surgery + payer		Patient-level + minimally invasive surgery + payer + Black serving		Patient-level + minimally invasive surgery + payer + Black serving + surgeon volume	
	OR (95% CI)	*P* value	OR (95% CI)	*P* value	OR (95% CI)	*P* value	OR (95% CI)	*P* value	OR (95% CI)	*P* value	OR (95% CI)	*P* value
Race and ethnicity												
Non-Hispanic White	1 [Reference]	NA	1 [Reference]	NA	1 [Reference]	NA	1 [Reference]	NA	1 [Reference]	NA	1 [Reference]	NA
Non-Hispanic Black	1.62 (1.51-1.74)	<.001	1.25 (1.16-1.35)	<.001	1.25 (1.16-1.34)	<.001	1.22 (1.14-1.31)	<.001	1.14 (1.06-1.23)	.001	1.14 (1.06-1.23)	<.001
Hispanic	1.26 (1.09-1.45)	<.001	1.08 (0.95-1.22)	.246	1.09 (0.96-1.23)	.171	1.08 (0.96-1.21)	.227	1.07 (0.95-1.21)	.257	1.06 (0.95-1.18)	.325
Minimally invasive	NA	NA	NA	NA	0.85 (0.78-0.92)	<.001	0.86 (0.79-0.93)	<.001	0.86 (0.79-0.93)	<.001	1.01 (0.94-1.09)	.735
Payer												
Medicaid	NA	NA	NA	NA	NA	NA	1 [Reference]		1 [Reference]		1 [Reference]	
Self-paid	NA	NA	NA	NA	NA	NA	0.89 (0.78-1.02)	.107	0.88 (0.77-1.01)	.060	0.88 (0.77-1.01)	.074
Medicare	NA	NA	NA	NA	NA	NA	0.95 (0.87-1.04)	.303	0.95 (0.87-1.04)	.294	0.98 (0.9-1.07)	.727
HMO	NA	NA	NA	NA	NA	NA	0.85 (0.77-0.94)	.002	0.85 (0.77-0.94)	.002	0.91 (0.82-1.01)	.067
Other	NA	NA	NA	NA	NA	NA	0.94 (0.83-1.06)	.341	0.94 (0.83-1.06)	.313	0.95 (0.84-1.07)	.396
Commercial	NA	NA	NA	NA	NA	NA	0.8 (0.73-0.87)	<.001	0.8 (0.73-0.87)	<.001	0.84 (0.77-0.91)	<.001
Proportion of non-Hispanic Black, %												
<5	NA	NA	NA	NA	NA	NA	NA	NA	1 [Reference]		1 [Reference]	
5.0-9.9	NA	NA	NA	NA	NA	NA	NA	NA	1.06 (0.98-1.15)	.147	1.1 (1.02-1.19)	.019
10.0-19.9	NA	NA	NA	NA	NA	NA	NA	NA	1.02 (0.92-1.13)	.707	1.05 (0.96-1.14)	.287
20.0-20.9	NA	NA	NA	NA	NA	NA	NA	NA	1.22 (1.08-1.38)	.002	1.23 (1.11-1.38)	<.001
≥30.0	NA	NA	NA	NA	NA	NA	NA	NA	1.32 (1.13-1.54)	<.001	1.25 (1.09-1.44)	.001
Surgeon volume	NA	NA	NA	NA	NA	NA	NA	NA	NA			
<20	NA	NA	NA	NA	NA	NA	NA	NA	NA	NA	1 [Reference]	
20-49	NA	NA	NA	NA	NA	NA	NA	NA	NA	NA	0.81 (0.74-0.89)	<.001
50-99	NA	NA	NA	NA	NA	NA	NA	NA	NA	NA	0.69 (0.63-0.76)	<.001
100-199	NA	NA	NA	NA	NA	NA	NA	NA	NA	NA	0.61 (0.55-0.67)	<.001
200-299	NA	NA	NA	NA	NA	NA	NA	NA	NA	NA	0.55 (0.47-0.63)	<.001
≥300	NA	NA	NA	NA	NA	NA	NA	NA	NA	NA	0.43 (0.37-0.51)	<.001

Abbreviations: HMO, health maintenance organization; NA, not applicable; OR, odds ratio.

Surgeon case volume was associated with death or major complications. Patients treated by high-volume surgeons had 57% lower odds of death or major complications (aOR, 0.43; 95% CI, 0.37-0.51; *P* < .001) compared with patients treated by low-volume surgeons, after adjusting for patient risk, payer status, the use of a minimally invasive approach, and the hospital proportion of non-Hispanic Black individuals (Table 2). When treated by high-volume surgeons, non-Hispanic Black individuals were more likely to die or have a major complication (aOR, 0.57; 95% CI, 0.44-0.73) than non-Hispanic White individuals (aOR, 0.42; 95% CI, 0.36-0.50) compared with non-Hispanic White patients treated by low-volume surgeons (*P* = .02 for comparison of non-Hispanic Black with non-Hispanic White within same surgeon volume strata) (eTable 3 in Supplement 1).

## Discussion

Using national all-payer data on 103 753 patients undergoing mitral valve surgery at 1085 hospitals, we found that non-Hispanic Black individuals had 35% lower odds of undergoing a minimally invasive approach than non-Hispanic White individuals. This gap was no longer significant after adjusting for patient risk factors, payer status, and access to high-volume surgeons. Hispanic individuals were not less likely to receive a minimally invasive approach compared with non-Hispanic White individuals. Non-Hispanic Black individuals were also 62% more likely to die or experience a major complication than non-Hispanic White individuals, whereas Hispanic individuals were 26% more likely to experience death or a major complication than non-Hispanic White individuals.

Our findings demonstrate the extent of differential access to minimally invasive mitral valve surgery. These findings are unfortunately not surprising in light of the extensive evidence of racial and ethnic disparities in cardiovascular medicine.^3^ The American Heart Association recently endorsed the need to address structural racism by addressing uneven access to health insurance and quality medical care.^39^ Our findings have possible policy implications for promoting equity in cardiovascular care. We noted a number of patterns that may suggest potential mechanisms by which these inequities occur, and therefore suggest ways they might be addressed. We found that Black patients were much less likely to have private insurance than White patients, and that patients with private insurance were more likely to undergo minimally invasive surgery than patients with Medicaid or without insurance. Thus, reducing the number of uninsured individuals via Medicaid expansion alone may not reduce disparities in access to minimally invasive cardiac procedures. Instead, efforts to increase access to commercial insurance or expand Medicare coverage—as opposed to Medicaid expansion alone—may be more successful in promoting racial equity. Lowering the age of eligibility or creating a Medicare buy-in may reduce disparities.^40^ Of note, even among patients with commercial or Medicare insurance, non-Hispanic Black individuals were still less likely to undergo minimally invasive surgery than non-Hispanic White individuals. However, any policy solutions implied by our results should be considered hypothesis-generating; testing programmatic changes can best be evaluated using a randomized clinical trial as was done for Medicaid expansion in the Oregon experiment,^41^ and for bundled payments in the Comprehensive Care for Joint Replacement.^42,43^ While most policies are not evaluated in this manner, this degree of rigor may be necessary to ensure policies are optimally improving equity.

Second, the segregation of non-Hispanic Black individuals in high-minority hospitals likely contributes to disparities in the use of minimally invasive surgery. High-minority hospitals have fewer resources than hospitals that serve a lower proportion of individuals from racial and ethnic minority groups,^44^ and are more often penalized by CMS value-based purchasing programs.^45^ Although recent policy changes have attempted to mitigate these disproportionate penalties,^46^ efforts to improve racial equity may require CMS to shift resources toward hospitals that care for greater numbers of disadvantaged individuals, or to explicitly incentivize and reward providing access to care for patients who have been historically marginalized due to structural and systemic racism. Groups such as US News and World Report and the Lown Group have recently published hospital equity performance measures that begin to examine these areas,^47,48^ but more work is needed to create, validate, and apply better measures of equitable access to important procedures and technologies.

Finally, given the striking volume-outcome association for mitral valve surgery, increasing access to high-volume operators is essential. This may be more challenging in rural areas, particularly rural areas that are disproportionately communities of racial and ethnic minority groups. However, a systems approach to health care, which prioritizes regionalizing care in areas where the volume-outcome association is particularly strong, might help improve these patterns. Patient education is also crucial, so that patients can make more informed decisions and advocate for themselves in terms of seeking care that is as optimal as possible.

### Limitations

Our study has several limitations. First, we chose to present the results of our unadjusted analyses as our main findings because health disparities begin long before surgery. Black individuals have worse cardiovascular health overall compared with non-Hispanic White individuals.^3^ Individuals from racial and ethnic minority groups face social and structural barriers to preventive health resources, and excessive activation of the stress response caused by safety, socioeconomic concerns, and racial discrimination, which lead to worsening health over time—sometimes described as “weathering.”^3,39^ Controlling for preexisting health conditions caused partly by structural and interpersonal racism may unintentionally minimize the magnitude of disparities. Nonetheless, because it is also essential to understand the impact of the health care system on disparities, we also report our findings after controlling for a wide range of patient factors, payer status, and surgeon case volume. This approach allowed us to consider changes in health policy that may help promote greater equity.

Second, our analysis does not consider patient preferences for minimally invasive surgery. Non-Hispanic Black individuals may be less willing to undergo surgery using a relatively new minimally invasive approach if they trust their physician less than non-Hispanic White individuals. In particular, Black individuals may distrust the health care system because of historical traumas (ie, Tuskegee) and, perhaps more importantly, because of everyday challenges of navigating a health care system that treats Black and White individuals differently.^49,50,51^ However, our finding that both non-Hispanic Black and non-Hispanic White individuals had greater than 15-fold higher use of the minimally invasive approach when treated by high and very-high volume surgeons suggests that lack of physician trust may not be the dominant factor influencing the choice of a minimally invasive approach, and that access is a primary consideration.

Finally, our study focused on one very innovative change in cardiovascular care, and does not include other recent advances, such as the use of minimally invasive approaches for aortic valve surgery, aortic surgery, and percutaneous approaches to aortic and mitral valve surgery. It will be important to examine disparities in these areas before drawing more definitive conclusions on differential access to some of the most innovative approaches in cardiovascular medicine.

## Conclusions

In this cross-sectional national study, we found that non-Hispanic Black patients are less likely to undergo minimally invasive mitral valve surgery and are more likely to die or experience major complications after mitral valve surgery than non-Hispanic White patients. Hispanic patients had similar rates of minimally invasive surgery as non-Hispanic White patients. Non-Hispanic Black patients were also more likely to have Medicaid, receive treatment from low-volume surgeons, and be hospitalized in high-minority hospitals compared with non-Hispanic White patients—all factors associated with less use of a minimally invasive approach. Efforts to reduce inequity in cardiovascular medicine may need to focus on expanding insurance coverage beyond Medicaid expansion and increasing access to high-volume surgeons.

## Notes

Supplement 1. **eFigure 1.** Flow Diagram Describing Selection of Cases in the Analytic Cohort **eFigure 2.** Disparities in the Utilization of Minimally Invasive Surgery in Non-Hispanic Black Individuals **eTable 1.** Hospital and Surgeon Characteristics **eTable 2.** Utilization of Minimally Invasive Approach **eTable 3.** 30-Day Mortality and Morbidity

Supplement 2.
Data Sharing Statement
