# Supplementary material for: Racial and Ethnic Disparities in Access to Minimally Invasive Mitral Valve Surgery
Source: JAMA Netw Open. 2022 Dec 21;5(12):e2247968. doi: 10.1001/jamanetworkopen.2022.47968 (PMC9857175; doi:10.1001/jamanetworkopen.2022.47968)
Supplement: Supplement 1. — eFigure 1. Flow Diagram Describing Selection of Cases in the Analytic Cohort eFigure 2. Disparities in the Utilization of Minimally Invasive Surgery in Non-Hispanic Black Individuals eTable 1. Hospital and Surgeon Characteristics eTable 2. Utilization of Minimally Invasive Approach eTable 3. 30-Day Mortality and Morbidity [file jamanetwopen-e2247968-s001.pdf]

Supplemental Online Content

Glance LG, Joynt Maddox KE, Mazzefi M, et al. Racial and ethnic disparities in access to minimally invasive mitral valve surgery. *JAMA Netw Open.* 2022;5(12):e2247968. doi:10.1001/jamanetworkopen.2022.47968

eFigure 1. Flow Diagram Describing Selection of Cases in the Analytic Cohort

eFigure 2. Disparities in the Utilization of Minimally Invasive Surgery in Non-Hispanic Black Individuals

eTable 1. Hospital and Surgeon Characteristics

eTable 2. Utilization of Minimally Invasive Approach

eTable 3. 30-Day Mortality and Morbidity

This supplemental material has been provided by the authors to give readers additional information about their work.

© 2022 Glance LG et al. *JAMA Network Open*.

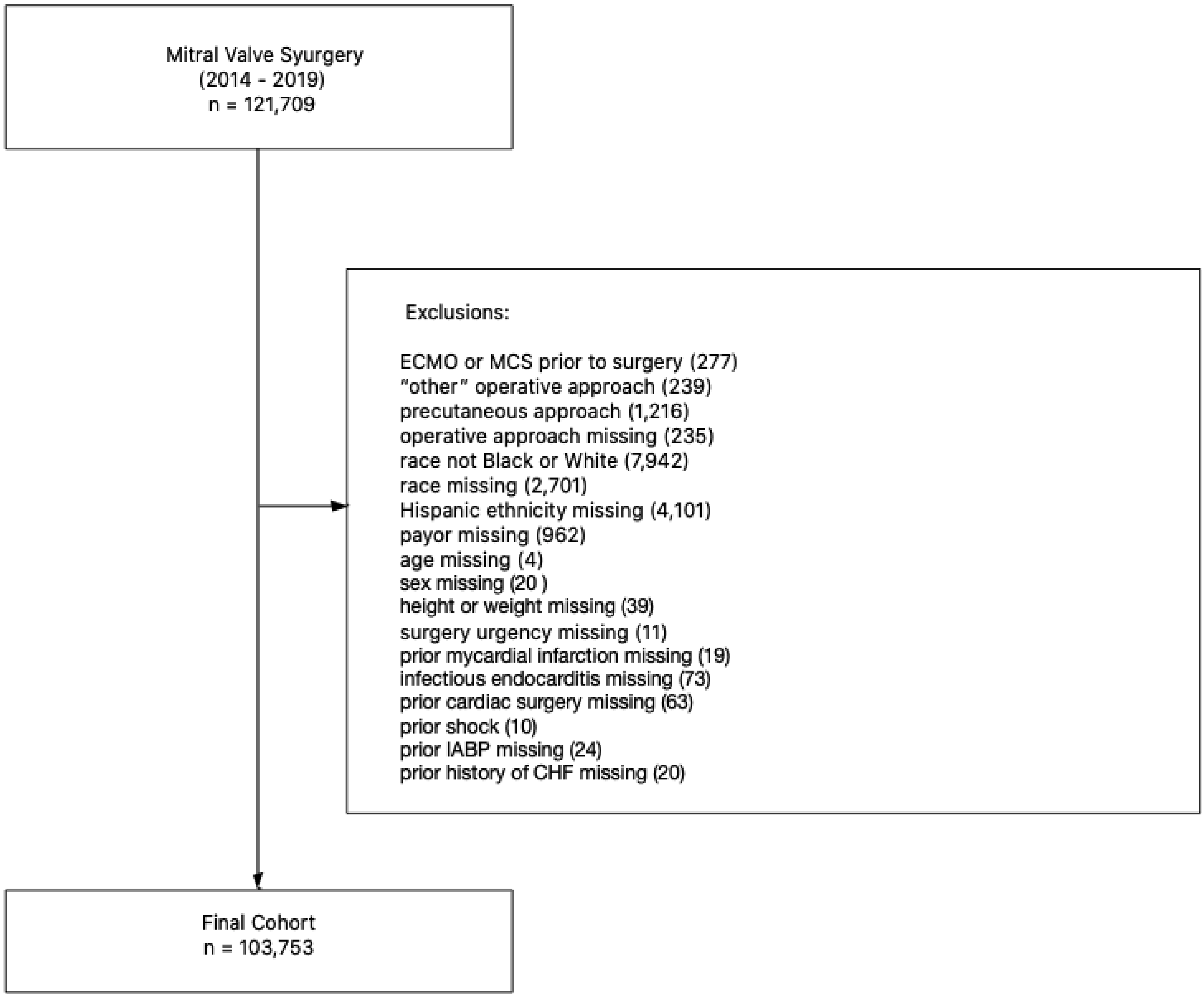

eFigure 1. Flow diagram describing selection of cases in the analytic cohort.

© 2022 Glance LG et al. *JAMA Network Open*.

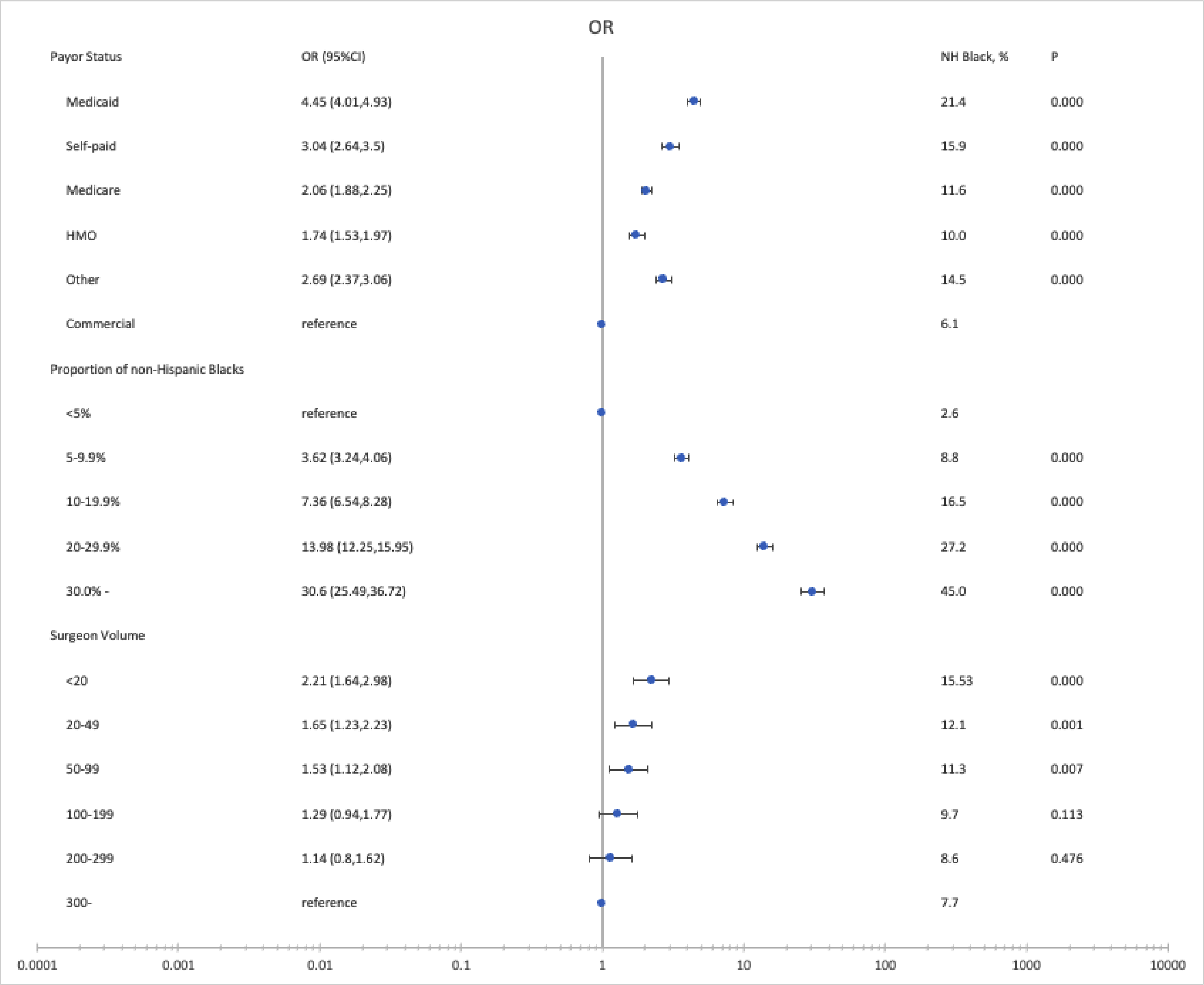

eFigure 2: Disparities in the utilization of minimally invasive surgery in non-Hispanic Black individuals. Association of non-Hispanic Black vs non-Hispanic White individuals versus payer status, hospital proportion of non-Hispanic Blacks, and surgeon case volume. Odds ratios were not adjusted except for payer status, which was adjusted for age.

© 2022 Glance LG et al. *JAMA Network Open*.

**eTable 1.** Hospital and surgeon characteristics.	
	Hospitals or Surgeons, No. (%)
Hospital proportion of non-Hispanic Blacks	
<5%	539 (49.7)
5-9.9%	194 (17.9)
10-19.9%	170 (15.7)
29-29.9%	78 (7.19)
>= 30%	104 (9.6)
Hospital region	
East South Central	74 (6.8)
Great Lakes	213 (19.6)
Middle Atlantic	89 (8.2)
Mountain	74 (6.8)
New England	35 (3.2)
Pacific	149 (13.7)
South Atlantic	176 (16.2)
West South Central	156 (14.4)
Missing	12 (1.1)
Surgeon case volume	
<20	1,248 (46.4)
20-49	713 (26.5)
50-99	410 (15.2)
100-199	212 (7.9)
200-299	62 (2.3)
>= 300	45 (1.7)

© 2022 Glance LG et al. *JAMA Network Open*.

eTable 2.Utilization of Minimally Invasive Approach								
	Unadjusted		Patient-level		Patient-level + payor		Patient-level + Payor + Black serving + Surgeon Volume	
	Odds Ratio (95% CI)	P value	Odds Ratio (95% CI)	P value	Odds Ratio (95% CI)	P value	Odds Ratio (95% CI)	P value
Race and ethnicity								
non-Hispanic White	reference		reference		reference		reference	
non-Hispanic Black	0.65 (0.58, 0.73)	<.001	0.88 (0.78, 0.99)	0.037	0.93 (0.82, 1.04)	0.212	0.99 (0.9, 1.09)	0.812
Hispanic	1.08 (0.67, 1.75)	0.738	1.36 (0.84, 2.19)	0.210	1.43 (0.89, 2.3)	0.134	1.59 (1.11, 2.29)	0.012
Payor								
Medicaid	reference		reference		reference		reference	
Self-paid	1.26 (1.02, 1.57)	0.034			1.23 (0.99, 1.53)	0.066	1.26 (1.02, 1.55)	0.034
Medicare	1.81 (1.6, 2.05)	<.001			1.4 (1.23, 1.59)	<.001	1.18 (1.05, 1.34)	0.008
HMO	1.74 (1.39, 2.19)	<.001			1.29 (1.02, 1.62)	0.032	0.95 (0.77, 1.16)	0.617
Other	1.35 (1.13, 1.6)	0.001			1.11 (0.93, 1.32)	0.252	1.04 (0.88, 1.24)	0.643
Commercial	2.35 (2.06, 2.68)	<.001			1.53 (1.37, 1.71)	<.001	1.23 (1.09, 1.38)	0.001
Proportion of non-Hispanic Black								
<5%	reference		reference		reference		reference	
5.0-9.9%	1.08 (0.77, 1.52)	0.650					0.98 (0.68, 1.39)	0.891
10.0-19.9%	1.1 (0.75, 1.61)	0.621					1.21 (0.81, 1.8)	0.352
20.0-29.9%	0.86 (0.56, 1.32)	0.490					1.03 (0.68, 1.57)	0.879
30.0% -	0.37 (0.23, 0.58)	<.001					0.59 (0.29, 1.22)	0.158
Surgeon volume								
<20	reference		reference		reference		reference	
20-49	2.41 (1.86, 3.13)	<.001					2.25 (1.73, 2.92)	<.001
50-99	4.41 (3.36, 5.77)	<.001					3.94 (3, 5.19)	<.001
100-199	9.26 (6.99, 12.27)	<.001					7.86 (5.91, 10.46)	<.001
200-299	21.23 (14.37, 31.35)	<.001					18.24 (12.17, 27.35)	<.001

© 2022 Glance LG et al. *JAMA Network Open*.

300-	20.72 (12.67, 33.9)	<.001					16.21 (9.84, 26.73)	<.001

	Unadjusted		Patient-level		Patient-level + payor		Patient-level + Payor + Black serving + Surgeon Volume	
	Odds Ratio (95% CI)	P value	Odds Ratio (95% CI)	P value	Odds Ratio (95% CI)	P value	Odds Ratio (95% CI)	P value
Age								
<40			reference		reference		reference	
41-50			1.08 (0.99, 1.18)	0.086	1.04 (0.95, 1.13)	0.403	1.07 (0.97, 1.18)	0.151
51-60			1.04 (0.95, 1.14)	0.352	0.99 (0.9, 1.08)	0.826	1.05 (0.95, 1.16)	0.318
61-70			0.98 (0.89, 1.07)	0.623	0.93 (0.84, 1.04)	0.207	0.98 (0.88, 1.1)	0.749
71-80			0.93 (0.82, 1.05)	0.232	0.9 (0.78, 1.04)	0.150	0.97 (0.84, 1.11)	0.646
81 -			0.97 (0.82, 1.16)	0.762	0.94 (0.77, 1.15)	0.545	0.99 (0.81, 1.2)	0.896
Sex								
male			reference		reference		reference	
female			0.82 (0.79, 0.86)	<0.001	0.83 (0.8, 0.86)	<0.001	0.82 (0.78, 0.86)	<0.001
BMI								
<18.5			0.89 (0.8, 0.99)	0.030	0.9 (0.81, 1)	0.057	0.9 (0.81, 1.01)	0.077
18.5-24.9			reference		reference		reference	
25-25.9			0.89 (0.86, 0.93)	<0.001	0.89 (0.86, 0.93)	<0.001	0.9 (0.86, 0.94)	<0.001
30-39.9			0.66 (0.62, 0.7)	<0.001	0.66 (0.62, 0.7)	<0.001	0.68 (0.64, 0.73)	<0.001
40 -			0.41 (0.36, 0.47)	<0.001	0.41 (0.36, 0.47)	<0.001	0.43 (0.37, 0.49)	<0.001
Surgical urgency								
Elective			reference		reference		reference	
Urgent			0.54 (0.46, 0.62)	<0.001	0.55 (0.47, 0.63)	<0.001	0.63 (0.54, 0.73)	<0.001
Emergent			0.24 (0.17, 0.35)	<0.001	0.25 (0.17, 0.35)	<0.001	0.35 (0.24, 0.51)	<0.001
Salvage			0.38 (0.14, 1)	0.051	0.38 (0.14, 1.02)	0.055	0.54 (0.18, 1.6)	0.262
Prior myocardial infarction								
none			reference		reference		reference	
> 21 days			1.09 (1, 1.19)	0.064	1.1 (1.01, 1.2)	0.034	1.09 (0.99, 1.2)	0.074
8-21 days			0.97 (0.73, 1.28)	0.817	0.98 (0.74, 1.29)	0.859	1.03 (0.79, 1.35)	0.832

© 2022 Glance LG et al. *JAMA Network Open*.

1-7 days			0.79 (0.59, 1.05)	0.103	0.79 (0.59, 1.06)	0.111	0.94 (0.7, 1.26)	0.682
< 24 hours			0.51 (0.24, 1.08)	0.080	0.51 (0.24, 1.06)	0.072	0.62 (0.28, 1.36)	0.232
	Unadjusted		Patient-level		Patient-level + payor		Patient-level + Payor + Black serving + Surgeon Volume	
	Odds Ratio (95% CI)	P value	Odds Ratio (95% CI)	P value	Odds Ratio (95% CI)	P value	Odds Ratio (95% CI)	P value
Aortic insufficiency								
None			reference		reference		reference	
Trivial			0.96 (0.89, 1.02)	0.190	0.95 (0.89, 1.02)	0.171	0.9 (0.84, 0.97)	0.003
Mild			0.9 (0.84, 0.97)	0.005	0.9 (0.84, 0.97)	0.005	0.87 (0.81, 0.93)	<0.001
Moderate			0.78 (0.68, 0.89)	<0.001	0.78 (0.69, 0.89)	<0.001	0.73 (0.64, 0.82)	<0.001
Severe			0.54 (0.31, 0.94)	0.028	0.54 (0.31, 0.94)	0.029	0.52 (0.28, 0.95)	0.035
Glomerular filtration rate (cc/min)								
Normal			reference		reference		reference	
60-89.9			1.01 (0.95, 1.08)	0.685	1.01 (0.95, 1.07)	0.776	0.98 (0.92, 1.05)	0.606
45-59.9			0.93 (0.86, 1.02)	0.111	0.93 (0.86, 1.02)	0.115	0.93 (0.86, 1.02)	0.111
30 to 44.9			0.91 (0.82, 1.01)	0.085	0.91 (0.82, 1.01)	0.088	0.91 (0.82, 1.01)	0.093
15 to 29.9			0.83 (0.7, 0.99)	0.033	0.83 (0.7, 0.98)	0.030	0.82 (0.69, 0.98)	0.028
Kidney failure			0.71 (0.61, 0.83)	<0.001	0.72 (0.62, 0.84)	<0.001	0.75 (0.63, 0.88)	<0.001
Lung disease								
none			reference		reference		reference	
Mild			0.89 (0.78, 1.01)	0.067	0.9 (0.79, 1.02)	0.104	0.98 (0.86, 1.11)	0.718
Moderate			0.83 (0.73, 0.94)	0.003	0.84 (0.74, 0.95)	0.007	0.95 (0.83, 1.08)	0.429
Severe			0.8 (0.69, 0.93)	0.005	0.82 (0.7, 0.95)	0.011	0.92 (0.79, 1.07)	0.259
Pneumonia								
None			reference		reference		reference	
Remote			0.98 (0.89, 1.07)	0.619	0.98 (0.9, 1.08)	0.697	0.93 (0.84, 1.03)	0.185
Recent			0.7 (0.62, 0.8)	<0.001	0.71 (0.62, 0.8)	<0.001	0.76 (0.66, 0.87)	<0.001
Stroke								
None			reference		reference		reference	

© 2022 Glance LG et al. *JAMA Network Open*.

>30 days			0.81 (0.75, 0.87)	<0.001	0.82 (0.76, 0.88)	<0.001	0.83 (0.77, 0.9)	<0.001
<= 30 days			0.66 (0.55, 0.79)	<0.001	0.67 (0.56, 0.8)	<0.001	0.73 (0.6, 0.88)	0.001
Liver disease			0.77 (0.69, 0.85)	<0.001	0.8 (0.73, 0.89)	<0.001	0.81 (0.73, 0.9)	<0.001
	Unadjusted		Patient-level		Patient-level + payor		Patient-level + Payor + Black serving + Surgeon Volume	
	Odds Ratio (95% CI)	P value	Odds Ratio (95% CI)	P value	Odds Ratio (95% CI)	P value	Odds Ratio (95% CI)	P value
Atrial fibrillation			0.77 (0.72, 0.81)	<0.001	0.77 (0.72, 0.82)	<0.001	0.78 (0.73, 0.83)	<0.001
Mechanism for MR								
Myxomatous degenerative			reference		reference		reference	
Rheumatic			0.65 (0.57, 0.73)	<0.001	0.67 (0.59, 0.75)	<0.001	0.82 (0.73, 0.92)	0.001
Functional			0.72 (0.61, 0.84)	<0.001	0.73 (0.62, 0.86)	<0.001	0.79 (0.67, 0.94)	0.008
Infectious endocarditis			0.55 (0.49, 0.61)	<0.001	0.56 (0.51, 0.63)	<0.001	0.75 (0.67, 0.84)	<0.001
other			0.71 (0.6, 0.84)	<0.001	0.71 (0.6, 0.84)	<0.001	0.88 (0.75, 1.04)	0.129
missing			0.77 (0.64, 0.92)	0.005	0.77 (0.64, 0.93)	0.007	0.98 (0.83, 1.16)	0.797
Prior cardiac surgery								
None			reference		reference		reference	
Prior CABG			1.43 (1.21, 1.68)	<0.001	1.43 (1.22, 1.68)	<0.001	1.43 (1.2, 1.71)	<0.001
Prior valve surgery			0.71 (0.61, 0.83)	<0.001	0.71 (0.61, 0.83)	<0.001	0.59 (0.5, 0.69)	<0.001
Prior CABG & valve surgery			1.49 (1.23, 1.79)	<0.001	1.49 (1.23, 1.79)	<0.001	1.31 (1.07, 1.61)	0.009
IABP			0.45 (0.34, 0.6)	<0.001	0.45 (0.34, 0.61)	<0.001	0.47 (0.31, 0.73)	0.001
Year								
2014			reference		reference		reference	
2015			1.25 (1.09, 1.43)	0.001	1.25 (1.1, 1.43)	0.001	1.24 (1.05, 1.45)	0.010
2016			1.42 (1.22, 1.66)	<0.001	1.42 (1.22, 1.67)	<0.001	1.44 (1.2, 1.73)	<0.001
2017			1.63 (1.39, 1.9)	<0.001	1.61 (1.38, 1.88)	<0.001	1.62 (1.35, 1.94)	<0.001
2018			1.73 (1.48, 2.03)	<0.001	1.69 (1.44, 1.99)	<0.001	1.72 (1.43, 2.07)	<0.001
2019			1.7 (1.44, 2.01)	<0.001	1.66 (1.4, 1.98)	<0.001	1.76 (1.44, 2.14)	<0.001

© 2022 Glance LG et al. *JAMA Network Open*.

eTable 3. 30-day Mortality or Morbidity.

	Unadjusted		Patient-level		Patient-level + mini		Patient-level + mini + Payor		Patient-level + mini + Payor + Black serving		Patient-level + mini + Payor + Black serving + Surgeon Volume		Patient-level + mini + Payor + Black serving + Surgeon Volume X Race/Ethnicity	
	OR (95% CI)	P	OR (95% CI)	P	OR (95% CI)	P	OR (95% CI)	P	OR (95% CI)	P	OR (95% CI)	P	Odds Ratio (95% CI)	P value
Race and ethnicity														
non-Hispanic White	reference		reference		reference		reference		reference		reference		reference	
non-Hispanic Black	1.62 (1.51,1.74)	0.000	1.25 (1.16,1.35)	0.000	1.25 (1.16,1.34)	0.000	1.22 (1.14,1.31)	0.000	1.14 (1.06,1.23)	0.001	1.14 (1.06,1.23)	0.000		
Hispanic	1.26 (1.09,1.45)	0.000	1.08 (0.95,1.22)	0.246	1.09 (0.96,1.23)	0.171	1.08 (0.96,1.21)	0.227	1.07 (0.95,1.21)	0.257	1.06 (0.95,1.18)	0.325		
Minimally invasive					0.85 (0.78,0.92)	0.000	0.86 (0.79,0.93)	0.000	0.86 (0.79,0.93)	0.000	1.01 (0.94,1.09)	0.735		
Payor														
Medicaid							reference		reference		reference		reference	
Self-paid							0.89 (0.78,1.02)	0.107	0.88 (0.77,1.01)	0.060	0.88 (0.77,1.01)	0.074	0.88 (0.77,1.01)	0.684
Medicare							0.95 (0.87,1.04)	0.303	0.95 (0.87,1.04)	0.294	0.98 (0.9,1.07)	0.727	0.98 (0.9,1.07)	0.000
HMO							0.85 (0.77,0.94)	0.002	0.85 (0.77,0.94)	0.002	0.91 (0.82,1.01)	0.067	0.91 (0.82,1)	0.000
Other							0.94 (0.83,1.06)	0.341	0.94 (0.83,1.06)	0.313	0.95 (0.84,1.07)	0.396	0.95 (0.84,1.07)	0.016
Commercial							0.8 (0.73,0.87)	0.000	0.8 (0.73,0.87)	0.000	0.84 (0.77,0.91)	0.000	0.84 (0.77,0.91)	0.273
Proportion of non-Hispanic Black														
<5%									reference		reference		reference	
5.0-9.9%									1.06 (0.98,1.15)	0.147	1.1 (1.02,1.19)	0.019	1.1 (1.02,1.19)	0.016
10.0-19.9%									1.02 (0.92,1.13)	0.707	1.05 (0.96,1.14)	0.287	1.05 (0.96,1.14)	0.273
20.0-29.9%									1.22 (1.08,1.38)	0.002	1.23 (1.11,1.38)	0.000	1.24 (1.11,1.38)	0.000
30.0% -									1.32 (1.13,1.54)	0.000	1.25 (1.09,1.44)	0.001	1.27 (1.11,1.46)	0.001
Surgeon volume														
<20											reference			
20-49											0.81 (0.74,0.89)	0.000		
50-99											0.69 (0.63,0.76)	0.000		
100-199											0.61 (0.55,0.67)	0.000		
200-299											0.55 (0.47,0.63)	0.000		
300-											0.43 (0.37,0.51)	0.000		
Race/ethnicity X Sugeon Volume*														
<20														
non-Hispanic White													reference*	
non-Hispanic Black													0.98 (0.82,1.17)	0.812
Hispanic													1.15 (0.89,1.47)	0.286
20-49														
non-Hispanic White													0.8 (0.72,0.88)	
non-Hispanic Black													0.91 (0.78,1.07)	0.077
Hispanic													0.79 (0.65,0.97)	0.958

© 2022 Glance LG et al. *JAMA Network Open*.

	Unadjusted		Patient-level		Patient-level + mini		Patient-level + mini + Payor		Patient-level + mini + Payor + Black serving		Patient-level + mini + Payor + Black serving + Surgeon Volume		Patient-level + mini + Payor + Black serving + Surgeon Volume X Race/Ethnicity	
	OR (95% CI)	P	OR (95% CI)	P	OR (95% CI)	P	OR (95% CI)	P	OR (95% CI)	P	OR (95% CI)	P	Odds Ratio (95% CI)	P value
50-99														
non-Hispanic White													0.69 (0.62,0.76)	
non-Hispanic Black													0.74 (0.64,0.86)	0.269
Hispanic													0.64 (0.51,0.82)	0.586
100-199														
non-Hispanic White													0.59 (0.53,0.65)	
non-Hispanic Black													0.67 (0.56,0.8)	0.089
Hispanic													0.8 (0.6,1.06)	0.023
200-299														
non-Hispanic White													0.52 (0.45,0.61)	
non-Hispanic Black													0.74 (0.6,0.91)	0.000
Hispanic													0.57 (0.32,1.01)	0.768
300-														
non-Hispanic White													0.42 (0.36,0.5)	
non-Hispanic Black													0.57 (0.44,0.73)	0.015
Hispanic													0.37 (0.3,0.46)	0.289
Age														
<40			reference		reference		reference		reference		reference			
41-50			0.99 (0.88,1.11)	0.863	0.99 (0.88,1.12)	0.888	1.01 (0.9,1.14)	0.842	1.01 (0.9,1.14)	0.874	1.01 (0.89,1.14)	0.915	1.01 (0.89,1.14)	0.874
51-60			1.11 (1,1.22)	0.051	1.11 (1,1.22)	0.046	1.14 (1.03,1.27)	0.011	1.14 (1.03,1.26)	0.013	1.14 (1.03,1.26)	0.014	1.14 (1.03,1.26)	0.013
61-70			1.25 (1.13,1.38)	0.000	1.25 (1.13,1.38)	0.000	1.24 (1.12,1.38)	0.000	1.24 (1.12,1.38)	0.000	1.24 (1.12,1.38)	0.000	1.25 (1.12,1.39)	0.000
71-80			1.64 (1.48,1.83)	0.000	1.64 (1.48,1.83)	0.000	1.57 (1.4,1.76)	0.000	1.57 (1.4,1.76)	0.000	1.58 (1.41,1.77)	0.000	1.58 (1.41,1.78)	0.000
81 -			1.89 (1.66,2.15)	0.000	1.9 (1.67,2.16)	0.000	1.81 (1.58,2.07)	0.000	1.81 (1.58,2.07)	0.000	1.83 (1.6,2.09)	0.000	1.84 (1.61,2.1)	0.000
Sex														
male			reference		reference		reference		reference		reference			
female			1.05 (1,1.09)	0.051	1.04 (0.99,1.09)	0.086	1.03 (0.99,1.08)	0.173	1.03 (0.98,1.08)	0.204	1.03 (0.98,1.08)	0.220	1.03 (0.98,1.08)	0.231
BMI														
<18.5			1.17 (1.03,1.34)	0.020	1.17 (1.02,1.34)	0.024	1.16 (1.01,1.33)	0.032	1.16 (1.01,1.33)	0.031	1.16 (1.01,1.33)	0.035	0.86 (0.75,0.99)	0.036
18.5-24.9			reference		reference		reference		reference		reference		reference	
25-25.9			0.98 (0.92,1.03)	0.393	0.97 (0.92,1.03)	0.324	0.97 (0.92,1.03)	0.361	0.97 (0.92,1.03)	0.343	0.97 (0.92,1.03)	0.353	0.84 (0.73,0.97)	0.014
30-39.9			1.18 (1.11,1.25)	0.000	1.16 (1.1,1.23)	0.000	1.17 (1.1,1.24)	0.000	1.16 (1.1,1.23)	0.000	1.16 (1.09,1.23)	0.000	1 (0.87,1.15)	0.996
40 -			1.81 (1.65,1.98)	0.000	1.77 (1.62,1.95)	0.000	1.77 (1.61,1.94)	0.000	1.77 (1.61,1.94)	0.000	1.77 (1.61,1.94)	0.000	1.53 (1.31,1.78)	0.000
Surgical urgency														
Elective			reference		reference		reference		reference		reference			
Urgent			1.47 (1.38,1.57)	0.000	1.45 (1.36,1.55)	0.000	1.44 (1.35,1.54)	0.000	1.43 (1.34,1.53)	0.000	1.38 (1.29,1.47)	0.000	1.38 (1.29,1.47)	0.000
Emergent			5.06 (4.26,6.01)	0.000	4.94 (4.15,5.86)	0.000	4.96 (4.17,5.89)	0.000	4.95 (4.16,5.88)	0.000	4.59 (3.87,5.45)	0.000	4.6 (3.88,5.47)	0.000
Salvage			6.99 (3.44,14.2)	0.000	6.89 (3.37,14.1)	0.000	6.93 (3.37,14.26)	0.000	6.88 (3.35,14.14)	0.000	6.33 (3.08,13.02)	0.000	6.36 (3.09,13.08)	0.000

© 2022 Glance LG et al. *JAMA Network Open*.

	Unadjusted		Patient-level		Patient-level + mini		Patient-level + mini + Payor		Patient-level + mini + Payor + Black serving		Patient-level + mini + Payor + Black serving + Surgeon Volume		Patient-level + mini + Payor + Black serving + Surgeon Volume X Race/Ethnicity	
	OR (95% CI)	P	OR (95% CI)	P	OR (95% CI)	P	OR (95% CI)	P	OR (95% CI)	P	OR (95% CI)	P	Odds Ratio (95% CI)	P value
Transfer from other hospital														
None			reference		reference		reference		reference		reference		reference	
Transfer			1.19 (1.1,1.29)	0.000	1.19 (1.1,1.28)	0.000	1.18 (1.09,1.28)	0.000	1.19 (1.1,1.29)	0.000	1.22 (1.12,1.31)	0.000	1.21 (1.12,1.31)	0.000
Shock			2.09 (1.54,2.83)	0.000	2.08 (1.54,2.81)	0.000	2.07 (1.53,2.8)	0.000	2.07 (1.53,2.81)	0.000	2 (1.47,2.71)	0.000	2.01 (1.48,2.72)	0.000
Ejection fraction														
60-			reference		reference		reference		reference		reference		reference	
50-59.9%			1.02 (0.97,1.08)	0.383	1.02 (0.97,1.08)	0.390	1.02 (0.97,1.07)	0.435	1.02 (0.97,1.07)	0.526	1 (0.95,1.05)	0.952	1 (0.95,1.05)	0.972
40-49.9%			1.2 (1.12,1.3)	0.000	1.2 (1.11,1.29)	0.000	1.19 (1.11,1.29)	0.000	1.19 (1.1,1.28)	0.000	1.17 (1.08,1.26)	0.000	1.17 (1.08,1.26)	0.000
30-39.9%			1.27 (1.15,1.41)	0.000	1.26 (1.14,1.4)	0.000	1.25 (1.13,1.39)	0.000	1.25 (1.13,1.38)	0.000	1.22 (1.11,1.35)	0.000	1.22 (1.11,1.35)	0.000
20-29.9%			1.47 (1.27,1.7)	0.000	1.47 (1.27,1.7)	0.000	1.46 (1.26,1.69)	0.000	1.45 (1.25,1.67)	0.000	1.45 (1.26,1.68)	0.000	1.46 (1.26,1.69)	0.000
<20%			2.48 (1.76,3.49)	0.000	2.48 (1.77,3.49)	0.000	2.48 (1.76,3.48)	0.000	2.44 (1.73,3.45)	0.000	2.49 (1.77,3.52)	0.000	2.49 (1.76,3.51)	0.000
Prior myocardial infarction														
none			reference		reference		reference		reference		reference		reference	
> 21 days			1.09 (1.01,1.19)	0.031	1.09 (1.01,1.19)	0.029	1.09 (1.01,1.18)	0.037	1.09 (1.01,1.18)	0.036	1.09 (1.01,1.19)	0.028	1.1 (1.01,1.19)	0.026
8-21 days			1.53 (1.26,1.86)	0.000	1.53 (1.26,1.86)	0.000	1.53 (1.26,1.85)	0.000	1.52 (1.25,1.84)	0.000	1.53 (1.27,1.86)	0.000	1.54 (1.27,1.86)	0.000
1-7 days			1.54 (1.26,1.86)	0.000	1.53 (1.26,1.85)	0.000	1.52 (1.26,1.85)	0.000	1.52 (1.25,1.84)	0.000	1.49 (1.23,1.81)	0.000	1.48 (1.22,1.8)	0.000
>6 days. But < 24 hours			1.61 (0.93,2.79)	0.090	1.6 (0.92,2.77)	0.095	1.6 (0.92,2.8)	0.096	1.62 (0.93,2.81)	0.090	1.54 (0.86,2.74)	0.143	1.53 (0.86,2.72)	0.149
< 6 hours			1.33 (0.61,2.92)	0.474	1.31 (0.6,2.86)	0.503	1.31 (0.6,2.88)	0.496	1.31 (0.6,2.86)	0.503	1.26 (0.57,2.78)	0.566	1.27 (0.57,2.82)	0.554
CHF														
none			reference		reference		reference		reference		reference		reference	
> 2 weeks			1.21 (1.14,1.29)	0.000	1.22 (1.14,1.3)	0.000	1.21 (1.14,1.29)	0.000	1.21 (1.14,1.29)	0.000	1.22 (1.15,1.29)	0.000	1.22 (1.15,1.29)	0.000
<= 2 weeks			1.25 (1.17,1.33)	0.000	1.24 (1.16,1.33)	0.000	1.24 (1.16,1.33)	0.000	1.25 (1.17,1.33)	0.000	1.24 (1.16,1.32)	0.000	1.24 (1.16,1.32)	0.000
Aortic insufficiency														
None			reference		reference		reference		reference		reference		reference	
Trivial			0.99 (0.93,1.04)	0.640	0.98 (0.93,1.04)	0.598	0.99 (0.93,1.04)	0.616	0.99 (0.93,1.05)	0.707	1 (0.95,1.06)	0.909	1 (0.95,1.06)	0.896
Mild			1.09 (1.02,1.16)	0.007	1.08 (1.02,1.15)	0.009	1.08 (1.02,1.15)	0.011	1.08 (1.02,1.15)	0.009	1.09 (1.03,1.16)	0.004	1.09 (1.03,1.16)	0.003
Moderate			1.1 (0.98,1.23)	0.124	1.09 (0.97,1.22)	0.156	1.08 (0.97,1.22)	0.172	1.09 (0.97,1.22)	0.152	1.1 (0.98,1.24)	0.105	1.1 (0.98,1.24)	0.098
Severe			1.24 (0.77,2.01)	0.373	1.23 (0.76,1.99)	0.394	1.25 (0.77,2.02)	0.364	1.26 (0.78,2.03)	0.342	1.26 (0.79,2.01)	0.323	1.27 (0.8,2.02)	0.310
Glomerular filtration rate														
Normal			reference		reference		reference		reference		reference		reference	
Mildly decreased (60-89.9)			1.04 (0.98,1.12)	0.213	1.04 (0.98,1.12)	0.204	1.04 (0.98,1.12)	0.203	1.05 (0.98,1.12)	0.180	1.05 (0.99,1.13)	0.127	1.05 (0.99,1.13)	0.123
Mildly to moderately decreased (45-59.9)			1.35 (1.24,1.46)	0.000	1.35 (1.24,1.46)	0.000	1.34 (1.24,1.45)	0.000	1.34 (1.24,1.45)	0.000	1.35 (1.24,1.46)	0.000	1.35 (1.24,1.46)	0.000
Moderately to severely decreased (30 to 44.9)			1.83 (1.67,2)	0.000	1.82 (1.67,2)	0.000	1.81 (1.66,1.98)	0.000	1.81 (1.66,1.99)	0.000	1.81 (1.66,1.98)	0.000	1.81 (1.66,1.98)	0.000
Severely decreased (15 to 29.9)			3.19 (2.79,3.65)	0.000	3.18 (2.78,3.63)	0.000	3.15 (2.76,3.6)	0.000	3.15 (2.76,3.6)	0.000	3.19 (2.79,3.64)	0.000	3.19 (2.8,3.65)	0.000
Kidney failure (<15)			3.28 (2.94,3.67)	0.000	3.26 (2.92,3.65)	0.000	3.15 (2.82,3.53)	0.000	3.14 (2.81,3.51)	0.000	3.14 (2.81,3.51)	0.000	3.14 (2.81,3.51)	0.000

© 2022 Glance LG et al. *JAMA Network Open*.

	Unadjusted		Patient-level		Patient-level + mini		Patient-level + mini + Payor		Patient-level + mini + Payor + Black serving		Patient-level + mini + Payor + Black serving + Surgeon Volume		Patient-level + mini + Payor + Black serving + Surgeon Volume X Race/Ethnicity	
	OR (95% CI)	P	OR (95% CI)	P	OR (95% CI)	P	OR (95% CI)	P	OR (95% CI)	P	OR (95% CI)	P	Odds Ratio (95% CI)	P value
Lung disease														
none			reference		reference		reference		reference		reference		reference	
Mild			1.15 (1.06,1.24)	0.000	1.15 (1.06,1.23)	0.000	1.13 (1.05,1.22)	0.001	1.13 (1.05,1.22)	0.002	1.11 (1.03,1.19)	0.008	1.11 (1.03,1.19)	0.008
Moderate			1.3 (1.18,1.42)	0.000	1.29 (1.18,1.42)	0.000	1.28 (1.17,1.41)	0.000	1.28 (1.17,1.4)	0.000	1.25 (1.14,1.37)	0.000	1.25 (1.14,1.36)	0.000
Severe			1.52 (1.38,1.68)	0.000	1.52 (1.38,1.68)	0.000	1.5 (1.36,1.65)	0.000	1.49 (1.35,1.65)	0.000	1.46 (1.32,1.61)	0.000	1.46 (1.32,1.61)	0.000
Home oxygen														
none			reference		reference		reference		reference		reference		reference	
partial			1.28 (1.1,1.48)	0.001	1.27 (1.1,1.47)	0.002	1.26 (1.09,1.46)	0.002	1.27 (1.09,1.47)	0.002	1.27 (1.09,1.47)	0.002	1.27 (1.1,1.47)	0.002
oxygen dependent			1.57 (1.35,1.83)	0.000	1.57 (1.35,1.82)	0.000	1.56 (1.34,1.81)	0.000	1.56 (1.34,1.81)	0.000	1.6 (1.38,1.86)	0.000	1.6 (1.37,1.86)	0.000
Pneumonia														
None			reference		reference		reference		reference		reference		reference	
Remote			1.05 (0.97,1.14)	0.229	1.05 (0.97,1.13)	0.235	1.05 (0.97,1.13)	0.263	1.05 (0.97,1.14)	0.229	1.06 (0.98,1.15)	0.122	1.07 (0.98,1.15)	0.117
Recent			1.55 (1.43,1.68)	0.000	1.54 (1.42,1.67)	0.000	1.54 (1.42,1.67)	0.000	1.54 (1.42,1.67)	0.000	1.52 (1.4,1.64)	0.000	1.52 (1.4,1.64)	0.000
Stroke														
None			reference		reference		reference		reference		reference		reference	
>30 days			1.19 (1.1,1.28)	0.000	1.18 (1.1,1.27)	0.000	1.17 (1.09,1.26)	0.000	1.17 (1.08,1.26)	0.000	1.16 (1.08,1.26)	0.000	1.17 (1.08,1.26)	0.000
<= 30 days			1.73 (1.53,1.95)	0.000	1.72 (1.52,1.94)	0.000	1.71 (1.52,1.93)	0.000	1.71 (1.52,1.94)	0.000	1.69 (1.5,1.91)	0.000	1.7 (1.5,1.91)	0.000
Liver disease			1.37 (1.25,1.5)	0.000	1.36 (1.24,1.49)	0.000	1.33 (1.21,1.46)	0.000	1.33 (1.21,1.46)	0.000	1.33 (1.21,1.46)	0.000	1.33 (1.21,1.46)	0.000
Atrial fibrillation			1.14 (1.08,1.2)	0.000	1.13 (1.08,1.19)	0.000	1.13 (1.08,1.19)	0.000	1.14 (1.08,1.2)	0.000	1.14 (1.08,1.2)	0.000	1.14 (1.08,1.2)	0.000
Peripheral vascular disease			1.18 (1.09,1.29)	0.000	1.18 (1.09,1.28)	0.000	1.17 (1.08,1.28)	0.000	1.17 (1.08,1.28)	0.000	1.18 (1.08,1.28)	0.000	1.18 (1.08,1.28)	0.000
Mechanism for MR														
Myxomatous degenerative			reference		reference		reference		reference		reference		reference	
Rheumatic			1.16 (1.07,1.27)	0.001	1.15 (1.06,1.26)	0.002	1.14 (1.04,1.24)	0.004	1.13 (1.04,1.24)	0.005	1.08 (0.99,1.17)	0.092	1.08 (0.99,1.17)	0.102
Functional			1.06 (0.92,1.21)	0.419	1.05 (0.92,1.2)	0.501	1.04 (0.91,1.19)	0.566	1.04 (0.91,1.18)	0.606	1.02 (0.9,1.17)	0.739	1.02 (0.89,1.16)	0.796
Infectious endocarditis			1.48 (1.37,1.6)	0.000	1.46 (1.35,1.58)	0.000	1.44 (1.33,1.56)	0.000	1.44 (1.33,1.56)	0.000	1.33 (1.23,1.44)	0.000	1.33 (1.23,1.44)	0.000
other			1.12 (1.04,1.21)	0.003	1.11 (1.03,1.2)	0.006	1.11 (1.03,1.2)	0.007	1.11 (1.03,1.2)	0.008	1.06 (0.98,1.14)	0.150	1.06 (0.98,1.14)	0.160
missing			1.13 (1.05,1.22)	0.002	1.12 (1.04,1.21)	0.003	1.12 (1.04,1.21)	0.004	1.12 (1.04,1.21)	0.003	1.06 (0.98,1.14)	0.152	1.05 (0.98,1.13)	0.165
Prior cardiac surgery														
None			reference		reference		reference		reference		reference		reference	
Prior CABG			1.37 (1.21,1.56)	0.000	1.39 (1.22,1.58)	0.000	1.39 (1.22,1.58)	0.000	1.38 (1.21,1.56)	0.000	1.39 (1.22,1.57)	0.000	1.39 (1.22,1.58)	0.000
Prior valve surgery			1.48 (1.37,1.6)	0.000	1.47 (1.37,1.59)	0.000	1.47 (1.36,1.59)	0.000	1.48 (1.37,1.59)	0.000	1.56 (1.44,1.68)	0.000	1.56 (1.45,1.68)	0.000
Prior CABG & valve surgery			1.61 (1.41,1.84)	0.000	1.63 (1.43,1.86)	0.000	1.63 (1.43,1.85)	0.000	1.62 (1.43,1.85)	0.000	1.69 (1.49,1.93)	0.000	1.69 (1.49,1.93)	0.000

© 2022 Glance LG et al. *JAMA Network Open*.

	Unadjusted		Patient-level		Patient-level + mini		Patient-level + mini + Payor		Patient-level + mini + Payor + Black serving		Patient-level + mini + Payor + Black serving + Surgeon Volume		Patient-level + mini + Payor + Black serving + Surgeon Volume X Race/Ethnicity	
	OR (95% CI)	P	OR (95% CI)	P	OR (95% CI)	P	OR (95% CI)	P	OR (95% CI)	P	OR (95% CI)	P	Odds Ratio (95% CI)	P value
Prior PCI														
none			reference		reference		reference		reference		reference		reference	
PCI, not acute			1.18 (1.09,1.28)	0.000	1.19 (1.09,1.29)	0.000	1.18 (1.09,1.28)	0.000	1.18 (1.08,1.28)	0.000	1.18 (1.09,1.28)	0.000	1.18 (1.09,1.28)	0.000
PCI, acute			1.51 (1.19,1.91)	0.000	1.53 (1.21,1.94)	0.000	1.53 (1.21,1.94)	0.000	1.53 (1.21,1.94)	0.000	1.52 (1.21,1.92)	0.000	1.52 (1.2,1.91)	0.000
IABP			3.45 (2.95,4.04)	0.000	3.4 (2.9,3.99)	0.000	3.41 (2.91,4)	0.000	3.4 (2.9,3.98)	0.000	3.36 (2.89,3.92)	0.000	3.37 (2.89,3.92)	0.000
Year														
2014			reference		reference		reference		reference		reference		reference	
2015			1.12 (1.03,1.23)	0.012	1.13 (1.03,1.23)	0.009	1.13 (1.03,1.23)	0.009	1.13 (1.03,1.23)	0.009	1.14 (1.04,1.25)	0.004	1.14 (1.04,1.25)	0.000
2016			1.19 (1.09,1.3)	0.000	1.2 (1.09,1.31)	0.000	1.2 (1.09,1.31)	0.000	1.19 (1.09,1.31)	0.000	1.2 (1.09,1.31)	0.000	1.2 (1.09,1.31)	0.000
2017			1.16 (1.06,1.27)	0.002	1.17 (1.07,1.28)	0.001	1.17 (1.07,1.28)	0.001	1.17 (1.07,1.28)	0.001	1.18 (1.07,1.29)	0.000	1.18 (1.07,1.29)	0.004
2018			1.14 (1.04,1.25)	0.006	1.15 (1.05,1.27)	0.002	1.15 (1.05,1.26)	0.003	1.15 (1.05,1.27)	0.003	1.16 (1.06,1.27)	0.002	1.16 (1.06,1.27)	0.000
2019			1.11 (1.01,1.22)	0.025	1.13 (1.03,1.24)	0.011	1.12 (1.02,1.24)	0.015	1.13 (1.02,1.24)	0.014	1.12 (1.02,1.23)	0.023	1.12 (1.02,1.23)	0.000

© 2022 Glance LG et al. *JAMA Network Open*.
