# Supplementary material for: Racial and Ethnic Disparities in Access to Minimally Invasive Mitral Valve Surgery
Source: JAMA Netw Open. 2022 Dec 21;5(12):e2247968. doi: 10.1001/jamanetworkopen.2022.47968 (PMC9857175; doi:10.1001/jamanetworkopen.2022.47968)
Supplement: Supplement 2. — Data Sharing Statement [file jamanetwopen-e2247968-s002.pdf]

Data Sharing Statement

Glance. Racial and Ethnic Disparities in Access to Minimally Invasive Mitral Valve Surgery. *JAMA Netw Open*. Published December 21, 2022. doi:10.1001/jamanetworkopen.2022.47968

Data
Data available: No

Additional Information
Explanation for why data not available: This data is non-public data obtained from the Society of Thoracic Surgeons and cannot be distributed as per the data use agreement.
